# Unlocking New Potential in the Functionalization of Chlorinated Silsesquioxanes: A Rapid and Chemoselective Thiolation Method

**DOI:** 10.3390/molecules30173583

**Published:** 2025-09-02

**Authors:** Niyaz Yagafarov, Yujia Liu, Naoto Adachi, Nobuhiro Takeda, Masafumi Unno, Armelle Ouali

**Affiliations:** 1 Department of Chemistry and Chemical Biology, Gunma University, 1-5-1 Tenjin-cho, Kiryu 376-8515, Japan; niiaz.iagafarov@gmail.com (N.Y.); t221a006@gunma-u.ac.jp (N.A.); ntakeda@gunma-u.ac.jp (N.T.); 2 ICGM, Univ Montpellier, CNRS, ENSCM, (Institut Charles Gerhardt Montpellier, Université de Montpellier, Centre National de la Recherche Scientifique, École Nationale Supérieure de Chimie de Montpellier), 1919 Route de Mende, CEDEX 05, 34293 Montpellier, France

**Keywords:** well-defined chlorinated silsesquioxanes, nucleophilic substitution, thiolation, mild organic transformation, click chemistry

## Abstract

A highly efficient method was successfully applied for the first time to the functionalization of well-defined chlorinated silsesquioxanes with a range of thiols. Thiolation was rapid (2 to 4 h), quantitative, with complete conversion of the reactants and full chemoselectivity, and proceeded under mild conditions (room temperature). This “click chemistry” approach facilitated the preparation of nine novel compounds, with good to excellent isolated yields (64–92%). The structures and purities of these compounds were comprehensively confirmed using multiple analytical techniques, including ^1^H, ^13^C, and ^29^Si NMR spectroscopy, elemental analysis, and mass spectrometry. Thermogravimetric analysis (TGA) further demonstrated that the synthesized compounds exhibited excellent thermal stability. These characteristics suggest their potential for applications in various domains of science, technology, and medicine.

## 1. Introduction

Silsesquioxanes (SQs) are hybrid organosilicon compounds with the general formula [RSiO_1.5_]_n_, where R represents hydrogen or various organic substituents. These compounds feature a stable and robust inorganic framework combined with reactive organic fragments, offering unique properties that make them suitable for a wide range of applications [1,2,3,4,5,6,7,8]. SQs with well-defined structures, such as cage SQs (Tn, *n* = 6, 8, 10,…,18) [3,9,10,11,12,13], open and closed double-decker SQs [14], and ladder SQs with varying numbers of fused rings [15], exhibit enhanced thermal stability and improved mechanical and electronic properties compared to their randomly structured counterparts. All-*cis*-cyclotetrasiloxanes, which feature a Janus-like structure with two distinct sides, have also drawn considerable interest due to their unique properties [16].

**Scheme 1:**
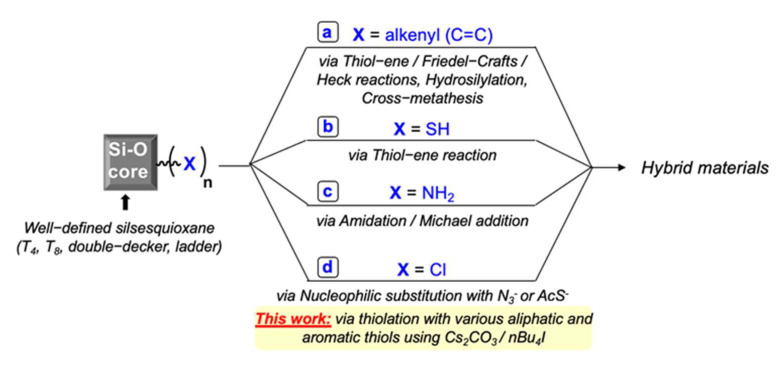
Functionalization of well-defined silsesquioxanes.

Among all these well-defined SQs, those with reactive functional groups stand out as promising building blocks for hybrid materials. Their ability to undergo various organic transformations allows for the introduction of diverse functional groups, expanding their potential applications (Scheme 1). For example, alkenyl-substituted SQs (Scheme 1, a, X = C=C) have been functionalized through a variety of organic reactions, including thiol-ene reaction [17,18,19,20,21,22,23,24,25,26,27,28,29,30,31,32,33,34,35,36,37,38], hydrosilylation [39,40,41,42,43,44,45,46,47,48,49], cross-metathesis [50,51,52,53,54,55,56,57], Heck couplings [52,54,58,59,60,61,62,63,64,65,66,67], and Friedel-Crafts reactions [68,69,70]. The resulting hybrid materials exhibited promise for use in coatings [17], flame retardants [18], catalysts/photocatalysts [20,44,67,70,71,72], membranes [21,24,41], sensors [22,25,42,49,59,60,70], biomedical applications [27,28,29,31,32], electronic devices [51,58,61,63,64,65], and more. Additionally, SQs bearing multiple thiol groups (Scheme 1, b, X = SH) can undergo efficient click thiol-ene addition reactions to form hybrids for applications such as protective coatings [73,74], tissue engineering [75,76,77,78], and fluorescent sensing [79,80,81]. SQs with amines (Scheme 1, c, X = NH_2_) are good candidates for amidation or Michael-type addition reactions, for example to prepare dendrimers [82,83,84,85]. An interesting class of functionalizable SQs consists of readily available chloro-substituted SQs (Scheme 1, d, X = Cl) which can undergo nucleophilic substitution by azide salts; the resulting azido-terminated SQs serve as versatile platform molecules for further applications, such as recyclable catalysts [44,86], bioactive compounds [87,88,89,90,91], and optoelectronic devices [92,93].

Moreover, chlorinated SQs can also react with thioacetates to yield thiolated SQs [35,94], as well as with a limited number of other reported thiolates [95,96,97]. One challenge in using azido or sulfur nucleophiles is the potential alteration of the SQ skeleton, which may undergo rearrangements [98].

In this study, we present an efficient method for the functionalization of well-defined chlorinated silsesquioxanes with various thiols. The thiolation process is rapid, quantitative, and selective, preserving the integrity of the SQ cage, and occurs under mild conditions. This approach, utilizing a cesium carbonate (Cs_2_CO_3_)-tetra-n-butylammonium iodide (TBAI) system, enables the straightforward preparation of thioether-functionalized SQs with high yields. It meets several criteria of a ‘click chemistry’ transformation, including mild conditions, complete conversion of the reactants in a very short time, full chemoselectivity, and modularity, as the method can be applied to various building blocks, potentially bearing functional groups.

To the best of our knowledge, this is the first application of this synthetic route for SQ functionalization, offering an efficient and versatile method for preparing SQ building blocks for hybrid materials.

## 2. Results and Discussion

**Scheme 2:**
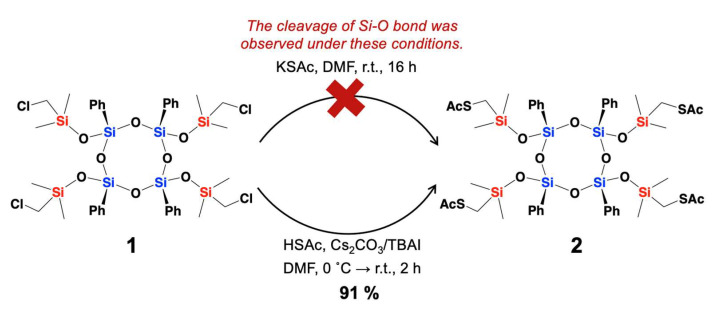
Attempts of synthesis of all-*cis*-T_4_ (**2**) using Cs_2_CO_3_/TBAI/HSAc system.

As mentioned earlier, an appealing strategy to prepare thioether-functionalized SQs is to carry out a nucleophilic substitution of chloro-terminated SQs with thiols. However, this approach is challenging, because the SQ skeleton can undergo undesired rearrangements in the presence of nucleophiles [98]. This is precisely what we observed in our attempts to thiolate the tetrachloro-substituted all-*cis*-T4 (**1**) [99] with potassium thioacetate (KSAc) (Scheme 2), according to strategies previously reported by some of us for other SQs [35,94]. These difficulties ultimately motivated the investigation described in this work.

In this study, we explored the use of cesium carbonate (Cs_2_CO_3_) and tetrabutylammonium iodide (TBAI) [100] for the thiolation of the challenging compound **1**. A solution of Cs_2_CO_3_ (2 equiv./Cl), TBAI (2 equiv./Cl), and thioacetic acid (HSAc, 2 equiv./Cl) in anhydrous DMF was stirred at room temperature for one hour before the dropwise addition of a solution of compound **1** in anhydrous DMF at 0 °C. After addition, the mixture was stirred for one hour while gradually warming to ambient temperature. Since HSAc is volatile, a straightforward work-up (extraction and evaporation) afforded pure compound **2** in an isolated yield of 91% (Scheme 2). Lower equivalents of Cs_2_CO_3_, TBAI, and HSAc were tested; however, complete conversion of compound **1** could not be achieved. The structure and purity of compound **2** were confirmed by multinuclear NMR spectroscopy (Figures S1–S3), mass spectrometry (Figure S38), and elemental analysis.

In the all-*cis* configuration (with all four phenyl groups on the same side), the four extended side chains are chemically equivalent. Therefore, in the ^29^Si NMR spectrum of **2** (Figure S3), two distinct signals were observed at −79.08 and 7.21 ppm, corresponding to the core Si atoms (T-unit Si, blue) and the Si atoms on the side chains (M-unit Si, red), respectively. In the ^1^H NMR spectrum (Figure S1), three singlets were observed at 0.28 ppm, 2.19 ppm, and 2.30 ppm, corresponding to the methyl groups attached to the M-unit Si atoms, the methylene groups adjacent to SAc, and the methyl groups of the acetyl group, respectively. The signals for the phenyl groups appeared in the range of 7.10–7.32 ppm.

The successful synthesis of compound **2** highlights the mildness and potential of the Cs_2_CO_3_/TBAI/thiol system for the thiolation of SQs. We then extended this approach to the tetrachloro all-*cis*-T_4_ bearing additional carbosilane linkers (compound **3**) [49]. Using similar conditions, but with lower equivalents of Cs_2_CO_3_, TBAI, and HSAc (1.1 equiv./Cl for each reagent), the target product (**4a**) was obtained after a simple work-up (without further purification) in high yield (91%). This may be attributed to the longer side chains of compound **3**, which increase molecular flexibility and may improve the accessibility of the chloro groups, thereby facilitating the reaction.

**Scheme 3:**
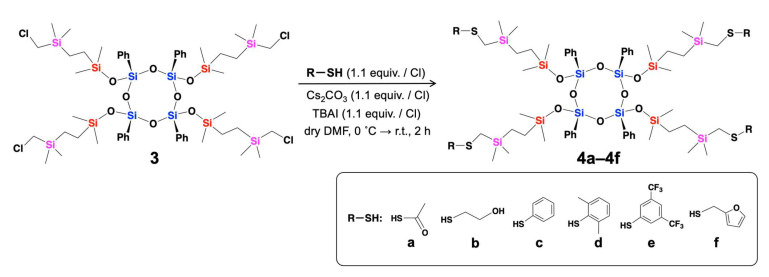
Synthesis of new functionalized all-*cis*-T_4_.

Subsequently, we explored the use of different commercially available thiols containing various functional groups, such as hydroxyl (OH), phenyl (Ph), trifluoromethyl (CF_3_), and furfuryl groups, in reactions with compound **3** under the same reaction conditions as those employed for the synthesis of **4a** (Scheme 3, Table 1).

The reaction with an aliphatic thiol containing a terminal unprotected OH (**b**) led to chemoselective S-alkylation, yielding compound **4b** in an isolated yield of 64% after extraction and evaporation. The relatively lower yield can be attributed to the partial partitioning of the product into the aqueous phase, which was observed to absorb UV light. At this stage, we did not attempt to optimize the procedure. This compound, which features four OH groups on the same face, could serve as a promising candidate for use as a surface-active agent [101].

Furthermore, reactions with aryl thiols bearing electron-donating and electron-withdrawing substituents (**c**–**e**) were performed under similar conditions. Since aryl thiols are less volatile, the removal of excess thiol required additional purification by column chromatography or gel permeation chromatography (GPC) after work-up. When using benzenethiol (**c**) and even a sterically hindered aromatic thiol (**d**), the corresponding products (**4c** and **4d**) were obtained in good isolated yields of 81% and 77%, respectively. Thiol **e**, which introduces CF_3_ groups, which are highly relevant in medicinal chemistry [102], also reacted successfully, although with a relatively lower isolated yield of 65%. Additionally, the reaction with furfuryl mercaptan provided compound **4f**, incorporating aromatic heterocycles (furfuryl groups), in a high isolated yield of 83%. This compound may serve as a valuable ligand for the synthesis of metal complexes for catalytic applications [103].

Compound **4a**–**4f** were fully characterized by multinuclear NMR spectroscopy (Figures S4–S22), mass spectrometry (Figures S39–S44), and elemental analysis. For compound **4a**, previously prepared by nucleophilic substitution using KSAc, the spectroscopic data are in agreement with the literature [71], while compounds **4b**–**4f** are new compounds.

**Scheme 4:**
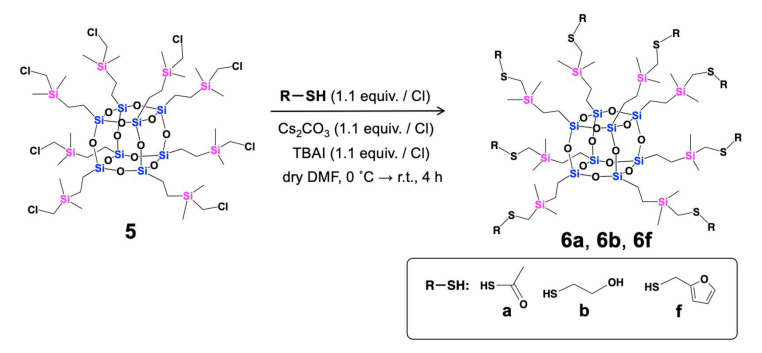
Synthesis of new functionalized T_8_ derivatives.

To further extend this synthetic method and demonstrate its applicability to other well-defined SQs, we selected three thiols with reactive groups (**a**, **b** and **f**) and reacted them with the reported octachloro-substituted cubic SQ (T_8_) (Scheme 4, Table 2) [44].

Using similar reaction conditions, a solution of compound **5** in anhydrous DMF was added dropwise to a pre-prepared mixture of Cs_2_CO_3_, TBAI, and HSAc (1.1 equiv. per Cl for each reagent) in anhydrous DMF at 0 °C. The reaction mixture was then stirred for up to three hours, gradually warming to room temperature, to achieve complete conversion of the eight chloro terminal substituents.

The target T_8_ derivatives (**6a** and **6b**) were successfully obtained in good isolated yields of 92% and 70%, respectively, after simple extraction and evaporation. Compound **6f** with furfuryl groups was isolated by column chromatography in 60% yield.

The newly synthesized thioether-containing T_8_ derivatives were thoughtfully characterized by multinuclear NMR spectroscopy (Figures S23–S31), mass spectrometry (Figures S45 and S46), and elemental analysis. For compound **6a**, previously prepared by nucleophilic substitution using KSAc, the spectroscopic data are in agreement with the literature [71], while compounds **6b** and **6c** are new compounds.

**Scheme 5:**
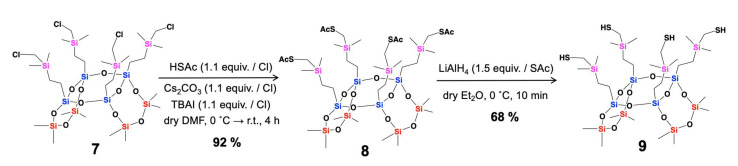
Synthesis of new functionalized tricyclic laddersiloxanes.

Moreover, this method was also successfully applied to the synthesis of a new thioacetate-substituted tricyclic laddersiloxane (**8**) from the laddersiloxane **7** [104]. The target compound **8** was obtained in excellent yield (92%) after a straightforward work-up. Subsequently, reduction of compound **8** with LiAlH_4_ afforded a new tetrathiol-substituted laddersiloxane (**9**) in good yield without any purification (68%, Scheme 5). This compound represents a promising precursor for the development of diverse hybrid materials.

In the ^29^Si NMR spectra of compounds **8** and **9** (Figures S34 and S37), three distinct signals were observed from right to left, corresponding to the T-unit Si atoms (blue), D-unit Si atoms (red), and Si atoms of the carbosilane moiety (pink), respectively. This pattern arises from the *syn*-type conformation, in which the four chains are located on one side and the two side rings on the opposite side. In the ^1^H NMR spectrum of compound **8** (Figure S32), three singlets observed at 0.05, 2.09, and 2.31 ppm were assigned to the methyl groups attached to the silicon atoms of the carbosilane, the methylene groups adjacent to SAc, and the methyl groups of the acetyl group. Two additional singlets observed at 0.09 and 0.13 ppm correspond to the methyl groups attached to the D-unit Si atoms. The two methyl groups on the same D-unit Si atoms are not chemically equivalent due to the *syn*-type structures [104]. The signals for the methylene groups between the T-unit Si atoms and the carbosilane appeared in the range of 0.48–0.57 ppm. In the ^1^H NMR spectrum of compound **9** (Figure S35), the signal for the acetyl methyl groups at 2.31 ppm disappeared, while a triplet for the SH group appeared at 1.10 ppm, and the signal for the methylene group adjacent to SH was observed as a doublet at 1.66 ppm. Compounds **8** and **9** were further characterized by ^13^C NMR spectroscopy (Figures S33 and S36), mass spectrometry (Figures S47 and S48), and elemental analysis.

The thermal properties of the synthesized thioether-containing SQs were investigated, revealing that all compounds exhibit high thermal stability (Table 3, Figures S49–S51). Specifically, compounds **2**, **6a**, **8**, and **9**, which bear relatively small thioether moieties, demonstrated particularly high stability, with decomposition temperatures at 5% weight loss (Td_5_) exceeding 300 °C—and in the case of compound **6a**, even exceeding 350 °C. The remaining compounds showed somewhat lower Td_5_ values, ranging from 207 °C to 253 °C. These results suggest that the thermal stability of these materials depends more strongly on the nature of the thioether substituents than on the structure of the siloxane core. The temperature of maximum decomposition rate (T_max_) of most samples exceeds 350 °C, except for compounds **4f** and **6f** (T_max_ = 323 and 315 °C, respectively), due to the lower thermal stability of the furyl moieties. Notably, all SQ thioethers displayed sublimation temperatures above 1000 °C, highlighting their remarkable thermal robustness and suitability for processing under extremely high-temperature conditions. It is worth noting that at 1000 °C, compound **6a** showed a residue of 48%, which is significantly higher than that of the other compounds. This can be explained by its structure: a higher proportion of thermally robust Si-O bonds and a lower proportion of organic moieties that decompose more easily under heat.

## 3. Materials and Methods

### 3.1. General Considerations

All reactions were performed under an argon atmosphere using the standard Schlenk technique. Diethyl ether, DMF, THF, and toluene were dried using the mBRAUN purification system. Triethylamine was distilled from potassium hydroxide and stored in potassium hydroxide under an argon atmosphere. Karstedt’s catalyst (in xylene, 2% Pt) was purchased from Sigma-Aldrich (St. Louis, MO, USA), (chloromethyl)dimethylsilane, benzenethiol, thioacetic acid, 3,5-Bis(trifluoromethyl)benzenethiol, and furfuryl mercaptan were purchased from TCI (Tokyo, Japan), cesium carbonate (Cs_2_CO_3_) was purchased from Kanto Chemical Co. Inc. (Tokyo, Japan), tetrabutylammonium iodide (TBAI) was purchased from FUJIFILM Wako Pure Chemical Co. (Tokyo, Japan), 2-mercaptoethanol was purchased from Kishida Chemical Co., Ltd. (Osaka, Japan), and 2,6-dimethylbenezenethiol was purchased from Acros Organics; all these reagents were used as received, without further purification. Fourier transformation nuclear magnetic resonance (NMR) spectra were obtained using a JEOL JNM-ECA 600 (^1^H at 600.17 MHz, ^13^C at 150.91 MHz, ^29^Si at 119.24 MHz, ^19^F at 564.46 MHz) NMR instrument. For ^1^H NMR, chemical shifts are reported as δ units (ppm) relative to SiMe_4_ (TMS), and residual solvent peaks were used as standards. For ^13^C NMR, ^29^Si NMR, and ^19^F NMR, chemical shifts are reported as δ units (ppm) relative to SiMe_4_ (TMS), the residual solvent peaks were used as standards, and spectra were obtained with complete proton decoupling. MALDI-TOF mass analysis was carried out with a Shimadzu AXIMA Performance instrument using 2,5-dihydroxybenzoic acid (dithranol) as the matrix and AgNO_3_ as the ion source. All reagents were of analytical grade. Elemental analyses were performed at the Center for Material Research by Instrumental Analysis (CIA), Gunma University, Japan. FTIR spectra were measured using a Shimadzu compact IRSpirit FTIR spectrophotometer. TGA was performed under a nitrogen flow (250 mL min^−1^) with a heating rate 10 °C min^−1^. All samples were measured at temperatures ranging from 50 to 1000 °C, where they remained for 5 min. The weight loss and heating rate were continuously recorded throughout the experiment.

### 3.2. Experimental Procedures for Compounds ***2***, ***4a***–***4f***, ***6a***, ***6b***, ***6f***, ***8***, and ***9***

a. Synthetic Procedure for Compound **2**

After standard evacuation and backfilling cycles with argon, an oven-dried Schlenk flask equipped with a magnetic stirring bar was charged with Cs_2_CO_3_ (0.26 g, 0.80 mmol, 8 equiv), TBAI (0.30 g, 0.80 mmol, 8 equiv), DMF (0.5 mL), and thioacetic acid (57 μL, 0.80 mmol, 8 equiv), and the mixture was stirred for 1 h at room temperature. After this time period, the reaction mixture was cooled to 0 °C and compound **1** (0.1 g, 0.1 mmol, 1 equiv) in DMF (0.5 mL) was added dropwise. The resulting mixture was stirred for an additional 1 h with gradual warming to ambient temperature. The resultant suspension was then poured into water (10 mL) and extracted with EtOAc (3 × 10 mL). The combined organic layers were washed with water (3 × 10 mL) and brine (10 mL), dried over sodium sulfate, concentrated on the rotavapor, and dried under vacuum to afford the desired pure product **2** (0.1 g, 91%) as a brown viscous oil without any purification.

b. General Synthetic Procedure for Compounds **4a**–**4f**

After standard evacuation and backfilling cycles with argon, an oven-dried Schlenk flask equipped with a magnetic stirring bar was charged with Cs_2_CO_3_ (0.11 g, 0.33 mmol, 4.4 equiv), TBAI (0.12 g, 0.33 mmol, 4.4 equiv), DMF (0.2 mL), and thiol (0.33 mmol, 4.4 equiv), and the mixture was stirred for 1 h at room temperature. After this time period, the reaction mixture was subsequently cooled to 0 °C, and compound **3** (0.1 g, 0.076 mmol, 1 equiv) in DMF (0.2 mL) was added dropwise. The resulting mixture was stirred for an additional 1 h with gradual warming to ambient temperature. The resultant suspension was then poured into water (10 mL) and extracted with EtOAc (3 × 10 mL). The combined organic layers were washed with water (3 × 10 mL) and brine (10 mL), dried over sodium sulfate, evaporated on the rotavapor, and dried under vacuum to afford the crude products.

The crude products were isolated as follows: compounds **4a** (brown viscous oil, 0.102 g, 91%) and **4b** (colorless viscous oil, 0.071 g, 64%) were obtained as pure products without further purification; compound **4c** (colorless viscous oil, 0.099 g, 81%) was purified by column chromatography (silica, hexane/EtOAc 5/1); and compounds **4d** (colorless viscous oil, 0.101 g, 77%), **4e** (colorless viscous oil, 0.109 g, 65%), and **4f** (brown viscous oil, 0.103 g, 83%) were purified by gel permeation chromatography (GPC).

c. General Synthetic Procedure for Compounds **6a**, **6b**, and **6f**

After standard evacuation and backfilling cycles with argon, an oven-dried Schlenk flask equipped with a magnetic stirring bar was charged with Cs_2_CO_3_ (0.58 g, 1.76 mmol, 8.8 equiv), TBAI (0.65 g, 1.76 mmol, 8.8 equiv), DMF (0.5 mL), and thiol (1.76 mmol, 8.8 equiv), and the mixture was stirred for 1 h at room temperature. After this time period, the reaction mixture was subsequently cooled to 0 °C and compound **5** (0.3 g, 0.2 mmol, 1 equiv) in DMF (0.5 mL) was added dropwise. The resulting mixture was stirred for an additional 3 h with gradual warming to ambient temperature. The resultant suspension was then poured into water (30 mL) and extracted with EtOAc (3 × 30 mL). The combined organic layers were washed with water (3 × 30 mL) and brine (10 mL), dried over sodium sulfate, evaporated on the rotavapor, and dried under vacuum to afford the crude products.

The crude products were isolated as follows: compounds **6a** (brown solid, 0.33 g, 92%) and **6b** (white solid, 0.26 g, 70%) were obtained as pure products without further purification; compound **6f** (yellow viscous oil, 0.25 g, 60%) was purified by column chromatography (silica, hexane/EtOAc 5/1).

d. Synthetic Procedure for Compound **8**

After standard evacuation and backfilling cycles with argon, an oven-dried Schlenk flask equipped with a magnetic stirring bar was charged with Cs_2_CO_3_ (0.15 g, 0.42 mmol, 4.4 equiv), TBAI (0.16 g, 0.42 mmol, 4.4 equiv), DMF (0.2 mL), and thioacetic acid (30 uL, 0.42 mmol, 4.4 equiv), and the mixture was stirred for 1 h at room temperature. After this time period, the reaction mixture was subsequently cooled to 0 °C and compound **7** (0.1 g, 0.096 mmol, 1 equiv) in DMF (0.2 mL) was added dropwise. The resulting mixture was stirred for an additional 3 h with gradual warming to ambient temperature. The resultant suspension was then poured into water (10 mL) and extracted with EtOAc (3 × 10 mL). The combined organic layers were washed with water (3 × 10 mL) and brine (10 mL), dried over sodium sulfate, evaporated on the rotavapor, and dried under vacuum to afford the desired pure product **8** (0.11 g, 92%) as a brown viscous oil without any purification.

e. Synthetic Procedure for Compound **9**

After standard evacuation and backfilling cycles with argon, an oven-dried Schlenk flask equipped with a magnetic stirring bar was charged with lithium aluminum hydride (0.08 g, 2.22 mmol) and anhydrous diethyl ether (2.0 mL). The mixture was then cooled to 0 °C. A solution of compound **8** (0.45 g, 0.37 mmol) in anhydrous diethyl ether (1.0 mL) pre-prepared under extremely anhydrous conditions was added dropwise under argon at 0 °C. The resulting mixture was stirred at 0 °C for 10 min and then hydrochloric acid solution (freshly prepared, 2 M, 1.0 mL) was added dropwise at 0 °C. The organic layer was washed with water, dried over sodium sulfate, and evaporated on the rotavapor to afford the desired product **9** (0.26 g, 68%) as a colorless viscous oil without any further purification.

### 3.3. Characterization Data for Compounds ***2***, ***4a***–***4f***, ***6a***, ***6b***, ***6f***, ***8***, and ***9***

Compound **2**: ^1^H NMR (600.17 MHz, CDCl_3_): δ = 0.28 (s, 24H, Si-(C*H*_3_)_2_), 2.19 (s, 8H, Si-C*H*_2_-S), 2.30 (s, 12H, S-C(O)-C*H*_3_), 7.10–7.12 (m, 8H, C_Ar_-*H*), 7.28–7.32 (m, 12H, C_Ar_-*H*) ppm; ^13^C{^1^H} NMR (150.91 MHz, CDCl_3_): δ = 0.07 (Si-(*C*H_3_)_2_), 14.98 (Si-*C*H_2_-S), 30.20 (S-C(O)-*C*H_3_), 127.65 (*C*_Ar_), 130.22 (*C*_Ar_), 132.03 (*C*_Ar_), 134.04 (*C*_Ar_), 196.27 (S-*C*(O)-CH_3_) ppm; ^29^Si{^1^H} NMR (119.24 MHz, CDCl_3_): δ = −79.08 (T-unit Si), 7.21 (M-unit Si) ppm; MALDI-TOF MS (m/z): 1160.95 ([M+Na]^+^, calcd. 1160.10); 1175.93 ([M+K]^+^, calcd. 1175.11); Elemental analysis: Calcd for C_44_H_64_O_12_S_4_Si_8_: C = 46.44; H = 5.67; S = 11.27; Found: C = 46.45; H = 5.76; S = 11.93.

Compound **4a**: ^1^H NMR (600.17 MHz, CDCl_3_): δ = −0.03 (s, 24H, Si-(C*H*_3_)_2_), 0.20 (s, 24H, Si-(C*H*_3_)_2_), 0.43 (s, 16H, Si-(C*H*_2_)_2_-Si), 2.03 (s, 8H, Si-C*H*_2_-S), 2.31 (s, 12H, S-C(O)-C*H*_3_), 7.06–7.10 (m, 8H, C_Ar_-*H*), 7.24–7.29 (m, 12H, C_Ar_-*H*) ppm; ^13^C{^1^H} NMR (150.91 MHz, CDCl_3_): δ = −3.99 (Si-(*C*H_3_)_2_), −0.25 (Si-(*C*H_3_)_2_), 6.37 (Si-*C*H_2_-CH_2_-Si(T)), 9.89 (Si-CH_2_-*C*H_2_-Si(T)), 12.63 (Si-*C*H_2_-S), 30.27 (S-C(O)-*C*H_3_), 127.53 (*C*_Ar_), 129.91 (*C*_Ar_), 133.12 (*C*_Ar_), 134.07 (*C*_Ar_), 197.07 (S-*C*(O)-CH_3_) ppm; ^29^Si{^1^H} NMR (119.24 MHz, CDCl_3_): δ = −79.50 (T-unit Si), 4.65 (M-unit Si), 11.27 (carbosilane) ppm; MALDI-TOF MS (m/z): 1505.29 ([M+Na]^+^, calcd. 1505.40); Elemental analysis: Calcd for C_60_H_104_O_12_S_4_Si_12_: C = 48.60; H = 7.07; S = 8.65; Found: C = 48.15; H = 7.26; S = 9.16.

Compound **4b**: ^1^H NMR (600.17 MHz, CDCl_3_): δ = 0.00 (s, 24H, Si-(C*H*_3_)_2_), 0.20 (s, 24H, Si-(C*H*_3_)_2_), 0.44 (s, 16H, Si-(C*H*_2_)_2_-Si), 1.68 (s, 8H, Si-C*H*_2_-S), 2.44 (br. s, 4H, O*H*), 2.66 (t, *J* = 5.8 Hz, 8H, S-C*H*_2_-CH_2_-OH), 3.69 (t, *J* = 5.8 Hz, 8H, C*H*_2_-OH), 7.05–7.08 (m, 8H, C_Ar_-*H*), 7.23–7.28 (m, 12H, C_Ar_-*H*) ppm; ^13^C{^1^H} NMR (150.91 MHz, CDCl_3_): δ = −3.94 (Si-(*C*H_3_)_2_), −0.26 (Si-(*C*H_3_)_2_), 6.38 (Si-*C*H_2_-CH_2_-Si(T)), 9.90 (Si-CH_2_-*C*H_2_-Si(T)), 15.79 (Si-*C*H_2_-S), 39.23 (S-*C*H_2_-CH_2_-OH), 59.13 (*C*H_2_-OH), 127.48 (*C*_Ar_), 129.85 (*C*_Ar_), 133.13 (*C*_Ar_), 134.06 (*C*_Ar_) ppm; ^29^Si{^1^H} NMR (119.24 MHz, CDCl_3_): δ = −79.52 (T-unit Si), 4.07 (M-unit Si), 11.33 (carbosilane) ppm; MALDI-TOF MS (m/z): 1512.33 ([M+Na]^+^, calcd. 1512.40); Elemental analysis: Calcd for C_60_H_112_O_12_S_4_Si_12_: C = 48.34; H = 7.57; S = 8.60; Found: C = 48.09; H = 7.15; S = 8.62.

Compound **4c**: ^1^H NMR (600.17 MHz, CDCl_3_): δ = 0.07 (s, 24H, Si-(C*H*_3_)_2_), 0.22 (s, 24H, Si-(C*H*_3_)_2_), 0.50 (s, 16 H, Si-(C*H*_2_)_2_-Si), 2.10 (s, 8H, Si-C*H*_2_-S), 7.04–7-11 (m, 12H, C_Ar_-*H*), 7.22–7.31 (m, 28H, C_Ar_-*H*) ppm; ^13^C{^1^H} NMR (150.91 MHz, CDCl_3_): δ = −3.75 (Si-(*C*H_3_)_2_), −0.20 (Si-(*C*H_3_)_2_), 6.58 (Si-*C*H_2_-CH_2_-Si(T)), 9.94 (Si-CH_2_-*C*H_2_-Si(T)), 16.51 (Si-*C*H_2_-S), 124.66 (*C*_Ar_), 126.06 (*C*_Ar_), 127.43 (*C*_Ar_), 128.76 (*C*_Ar_), 129.89 (*C*_Ar_), 133.18 (*C*_Ar_), 134.11 (*C*_Ar_), 140.63 (*C*_Ar_), ppm; ^29^Si{^1^H} NMR (119.24 MHz, CDCl_3_): δ = −79.46 (T-unit Si), 4.41 (M-unit Si), 11.29 (carbosilane) ppm; MALDI-TOF MS (m/z): 1641.09 ([M+Na]^+^, calcd. 1641.40); Elemental analysis: Calcd for C_76_H_112_O_8_S_4_Si_12_: C = 56.38; H = 6.97; S = 7.92; Found: C = 55.97; H = 6.70; S = 7.89.

Compound **4d**: ^1^H NMR (600.17 MHz, CDCl_3_): δ = 0.09 (s, 24H, Si-(C*H*_3_)_2_), 0.24 (s, 24H, Si-(C*H*_3_)_2_), 0.51 (s, 16H, Si-(C*H*_2_)_2_-Si), 1.90 (s, 8H, Si-C*H*_2_-S), 2.55 (s, 24H, C_Ar_-C*H*_3_), 7.05–7.11 (m, 20H, C_Ar_-*H*), 7.22–7.27 (m, 4H, C_Ar_-*H*), 7.30–7.32 (m, 8H, C_Ar_-*H*) ppm; ^13^C{^1^H} NMR (150.91 MHz, CDCl_3_): δ = −3.98 (Si-(*C*H_3_)_2_), −0.23 (Si-(*C*H_3_)_2_), 6.44 (Si-*C*H_2_-CH_2_-Si(T)), 10.02 (Si-CH_2_-*C*H_2_-Si(T)), 20.22 (Si-*C*H_2_-S), 21.89 (C_Ar_-*C*H_3_), 127.49 (*C*_Ar_), 127.74 (*C*_Ar_), 128.10 (*C*_Ar_), 129.84 (*C*_Ar_), 133.21 (*C*_Ar_), 134.09 (*C*_Ar_), 137.41 (*C*_Ar_), 142.44 (*C*_Ar_) ppm; ^29^Si{^1^H} NMR (119.24 MHz, CDCl_3_): δ = −79.48 (T-unit Si), 4.14 (M-unit Si), 11.37 (carbosilane) ppm; MALDI-TOF MS (m/z): 1753.54 ([M+Na]^+^, calcd. 1753.60); Elemental analysis: Calcd for C_84_H_128_O_8_S_4_Si_12_: C = 58.28; H = 7.45; S = 7.41; Found: C = 58.03; H = 7.46; S = 7.32.

Compound **4e**: ^1^H NMR (600.17 MHz, CDCl_3_): δ = 0.09 (s, 24H, Si-(C*H*_3_)_2_), 0.21 (s, 24H, Si-(C*H*_3_)_2_), 0.48–0.52 (m, 16H, Si-(C*H*_2_)_2_-Si), 2.10 (s, 8H, Si-C*H*_2_-S), 7.02–7.06 (m, 8H, C_Ar_-*H*), 7.20–7.29 (m, 12H, C_Ar_-*H*), 7.56 (s, 4H, C_Ar_-*H*), 7.59 (s, 8H, C_Ar_-*H*) ppm; ^13^C{^1^H} NMR (150.91 MHz, CDCl_3_): δ = −3.82 (Si-(*C*H_3_)_2_), −0.25 (Si-(*C*H_3_)_2_), 6.49 (Si-*C*H_2_-CH_2_-Si(T)), 9.86 (Si-CH_2_-*C*H_2_-Si(T)), 16.09 (Si-*C*H_2_-S), 118.04 (*C*_Ar_), 123.37 (q, *J* = 273.10 Hz, *C*F_3_), 125.07 (*C*_Ar_), 127.55 (*C*_Ar_), 129.98 (*C*_Ar_), 131.90 (q, *J* = 33.23 Hz, *C*_Ar_-CF_3_), 133.00 (*C*_Ar_), 134.06 (*C*_Ar_), 144.74 (*C*_Ar_) ppm; ^29^Si{^1^H} NMR (119.24 MHz, CDCl_3_): δ = −79.38 (T-unit Si), 4.78 (M-unit Si), 11.22 (carbosilane) ppm; ^19^F{^1^H} NMR (564.46 MHz, CDCl_3_): δ = −63.80 (C*F*_3_) ppm; MALDI-TOF MS (m/z): 2185.08 ([M+Na]^+^, calcd. 2185.30); Elemental analysis: Calcd for C_84_H_104_F_24_O_8_S_4_Si_12_: C = 46.65; H = 4.85; S = 5.93; Found: C = 47.83; H = 5.22; S = 6.38.

Compound **4f**: ^1^H NMR (600.17 MHz, CDCl_3_): δ = −0.01 (s, 24H, Si-(C*H*_3_)_2_), 0.20 (s, 24H, Si-(C*H*_3_)_2_), 0.43 (s, 16H, Si-(C*H*_2_)_2_-Si), 1.70 (s, 8H, Si-C*H*_2_-S), 3.67 (s, 8H, S-C*H*_2_-furyl), 6.16 (dd, *J* = 0.69 Hz, 4H, C_furyl_-*H*), 6.30 (dd, *J* = 1.83 Hz, 4H, C_furyl_-*H*), 7.06–7.10 (m, 8H C_Ar_-*H*), 7.24–7.30 (m, 12H, C_Ar_-*H*), 7.35 (dd, *J* = 0.92 Hz, 4H, C_furyl_-*H*) ppm; ^13^C{^1^H} NMR (150.91 MHz, CDCl_3_): δ = −3.86 (Si-(*C*H_3_)_2_), −0.24 (Si-(*C*H_3_)_2_), 6.47 (Si-*C*H_2_-CH_2_-Si(T)), 9.87 (Si-CH_2_-*C*H_2_-Si(T)), 16.69 (Si-*C*H_2_-S), 32.60 (S-*C*H_2_-furyl), 107.48 (*C*_furyl_), 110.40 (*C*_furyl_), 127.49 (*C*_Ar_), 129.82 (*C*_Ar_), 133.22 (*C*_Ar_), 134.10 (*C*_Ar_), 142.13 (*C*_furyl_), 151.94 (*C*_furyl_) ppm; ^29^Si{^1^H} NMR (119.24 MHz, CDCl_3_): δ = −79.54 (T-unit Si), 3.95 (M-unit Si), 11.37 (carbosilane) ppm; MALDI-TOF MS (m/z): 1657.4 ([M+Na]^+^, calcd. 1657.9); Elemental analysis: Calcd for C_52_H_96_O_8_S_4_Si_12_: C = 52.89; H = 6.91; S = 7.84; Found: C = 52.81; H = 6.99; S = 8.23.

Compound **6a**: ^1^H NMR (600.17 MHz, CDCl_3_): δ = 0.06 (s, 48H, Si-(C*H*_3_)_2_), 0.52–0.56 (m, 16H, Si-(C*H*_2_-CH_2_-Si(T)), 0.58–0.62 (m, 16H, Si-(CH_2_-C*H*_2_-Si(T)), 2.10 (s, 16H, C*H*_2_-SAc), 2.31 (s, 24H, S-C(O)-C*H*_3_) ppm; ^13^C{^1^H} NMR (150.91 MHz, CDCl_3_): δ = −3.86 (Si-(*C*H_3_)_2_), 4.37 (Si-*C*H_2_-CH_2_-Si(T)), 6.55 (Si-CH_2_-*C*H_2_-Si(T)), 12.61 (Si-*C*H_2_-S), 30.32 (S-C(O)-*C*H_3_), 196.94 (S-*C*(O)-CH_3_) ppm; ^29^Si{^1^H} NMR (119.24 MHz, CDCl_3_): δ = −66.61 (T-unit Si), 4.80 (carbosilane) ppm; MALDI-TOF MS (m/z): 1842.13 ([M+Na]^+^, calcd 1842.39), 1858.09 ([M+K]^+^, calcd 1858.49); Elemental analysis: Calcd for C_56_H_120_O_20_S_8_Si_16_: C = 36.97; H = 6.65; S = 14.10; Found: C = 36.98; H = 6.57; S = 13.51.

Compound **6b**: ^1^H NMR (600.17 MHz, CDCl_3_): δ = 0.09 (s, 48H, Si-(C*H*_3_)_2_), 0.55–0.60 (m, 16H, Si-(C*H*_2_-CH_2_-Si(T)), 0.61–0.66 (m, 16H, Si-(CH_2_-C*H*_2_-Si(T)), 1.73 (brs, 2H, O*H*), 1.79 (s, 16H, Si-C*H*_2_-S), 2.59 (brs, 6H, O*H*), 2.70 (t, *J* = 5.8 Hz, 16H, S-C*H*_2_-CH_2_-OH), 3.71–3.75 (m, 16H, C*H*_2_-OH) ppm; ^13^C{^1^H} NMR (150.91 MHz, CDCl_3_): δ = −3.78 (Si-(*C*H_3_)_2_), 4.43 (Si-*C*H_2_-CH_2_-Si(T)), 6.45 (Si-CH_2_-*C*H_2_-Si(T)), 16.04 (Si-*C*H_2_-S), 39.33 (S-*C*H_2_-CH_2_-OH), 59.31 (*C*H_2_-OH) ppm; ^29^Si{^1^H} NMR (119.24 MHz, CDCl_3_): δ = −66.49 (T-unit Si), 4.18 (carbosilane) ppm; MALDI-TOF MS (m/z): 1856.70 ([M+Na]^+^, calcd. 1856.36); 1874.68 ([M+K]^+^, calcd. 1874.33); Elemental analysis: Calcd for C_56_H_136_O_20_S_8_Si_16_: C = 36.64; H = 7.47; S = 13.97; Found: C = 36.53; H = 7.36; S = 13.44.

Compound **6f**: ^1^H NMR (600.17 MHz, CDCl_3_): δ = 0.05 (s, 48H, Si-(C*H*_3_)_2_), 0.49–0.53 (m, 16H, Si-(C*H*_2_-CH_2_-Si(T)), 0.59–0.63 (m, 16H, Si-(CH_2_-C*H*_2_-Si(T)), 1.77 (s, 16H, Si-C*H*_2_-S), 3.68 (s, 16H, S-C*H*_2_-furyl), 6.16–6.17 (m, 8H, C_furyl_-*H*), 6.29–6.30 (m, 8H, C_furyl_-*H*), 7.34–7.35 (m, 8H, C_furyl_-*H*) ppm; ^13^C{^1^H} NMR (150.91 MHz, CDCl_3_): δ = −3.75 (Si-(*C*H_3_)_2_), 4.38 (Si-*C*H_2_-CH_2_-Si(T)), 6.54 (Si-CH_2_-*C*H_2_-Si(T)), 16.72 (Si-*C*H_2_-S), 32.61 (S-*C*H_2_-furyl), 107.58 (*C*_furyl_), 110.41 (*C*_furyl_), 142.19 (*C*_furyl_), 151.88 (*C*_furyl_) ppm; ^29^Si{^1^H} NMR (119.24 MHz, CDCl_3_): δ = −66.47 (T-unit Si), 4.09 (carbosilane) ppm; Elemental analysis: Calcd for C_80_H_136_O_20_S_8_Si_16_: C = 45.24; H = 6.45; S = 12.08; Found: C = 44.61; H = 6.44; S = 12.41.

Compound **8**: ^1^H NMR (600.17 MHz, CDCl_3_): δ = 0.05 (s, 24H, Si-(C*H*_3_)_2_), 0.09 (s, 12H, Si(D)-(C*H*_3_)_2_), 0.13 (s, 12H, Si(D)-(C*H*_3_)_2_), 0.44–0.50 (m, 8H, Si-(C*H*_2_-CH_2_-Si(T)), 0.53–0.59 (m, 8H, Si-(CH_2_-C*H*_2_-Si(T)), 2.09 (s, 8H, Si-C*H*_2_-SAc), 2.31 (s, 12H, S-C(O)-C*H*_3_) ppm; ^13^C{^1^H} NMR (150.91 MHz, CDCl_3_): δ = −3.88 (Si-(*C*H_3_)_2_), 0.79 (Si (D)-(*C*H_3_)_2_), 0.92 (Si(D)-(*C*H_3_)_2_), 5.33 (Si-*C*H_2_-CH_2_-Si(T)), 6.65 (Si-CH_2_-*C*H_2_-Si(T)), 12.65 (Si-*C*H_2_-S), 30.29 (S-C(O)-*C*H_3_), 196.94 (S-*C*(O)-CH_3_) ppm; ^29^Si{^1^H} NMR (119.24 MHz, CDCl_3_): δ = −66.41 (T-unit Si), −18.66 (D-unit Si), 4.74 ppm (carbosilane); MALDI-TOF MS (m/z): 1228.79 ([M+Na]^+^, calcd.1228.20); 1243.71 ([M+K]^+^, calcd. 1243.20); Elemental analysis: Calcd for C_36_H_84_O_14_S_4_Si_12_: C = 35.84; H = 7.02; S = 10.63; Found: C = 36.44; H = 7.09; S = 10.78.

Compound **9**: ^1^H NMR (600.17 MHz, CDCl_3_): δ = 0.07 (s, 24H, Si-(C*H*_3_)_2_), 0.10 (s, 12H, Si(D)-(C*H*_3_)_2_), 0.15 (s, 12H, Si(D)-(C*H*_3_)_2_), 0.44–0.51 (m, 8H, Si-(C*H*_2_-CH_2_-Si(T)), 0.57–0.64 (m, 8H, Si-(CH_2_-C*H*_2_-Si(T)), 1.10 (t, *J* = 6.9 Hz, 4H, S*H*), 1.66 (d, *J* = 6.9 Hz, 8H, Si-C*H*_2_-SH) ppm; ^13^C{^1^H} NMR (150.91 MHz, CDCl_3_): δ = −4.45 (Si-(*C*H_3_)_2_), 0.79 (Si (D)-(*C*H_3_)_2_), 0.93 (Si(D)-(*C*H_3_)_2_), 5.35 (Si-*C*H_2_-CH_2_-Si(T)), 5.88 (Si-CH_2_-*C*H_2_-Si(T)), 6.59 (Si-*C*H_2_-SH) ppm; ^29^Si{^1^H} NMR (119.24 MHz, CDCl_3_): δ = −66.30 (T-unit Si), −18.71 (D-unit Si), 5.88 (carbosilane) ppm; MALDI-TOF MS (m/z): 1059.67 ([M+Na]^+^, calcd.1059.10); 1077.62 ([M+K]^+^, calcd. 1077.10); Elemental analysis: Calcd for C_28_H_76_O_10_S_4_Si_12_: C = 32.39; H = 7.38; S = 12.35; Found: C = 32.33; H = 7.42; S = 12.10.

## 4. Conclusions

In summary, we have developed a rapid, mild, and chemoselective synthetic method for the functionalization of well-defined chlorinated silsesquioxanes (SQs) featuring various cores (T_4_, T_8_, ladder-type), using a range of aliphatic and aromatic thiols. This thioether ligation strategy utilizes a Cs_2_CO_3_–TBAI system and enables the efficient preparation of a range of hybrid molecules with from good to excellent isolated yields (64–92%). The newly synthesized compounds were comprehensively characterized by multinuclear NMR spectroscopy, mass spectrometry, and elemental analysis. Thermogravimetric analysis (TGA) further confirmed their high thermal stability (with T_d5_ ranging from 207 to 350 °C). Overall, these thioether-functionalized SQs hold significant potential as building blocks for the design of novel hybrid materials. In addition, the convenient method reported herein for forming thioether linkages appears to be a versatile approach for functionalizing other chlorinated SQs to reach new functional materials.

## Figures and Tables

**Table 1:** Isolated yields of compounds **4a**–**4f**.

Compounds	Isolated Yield (%)
**4a**	91
**4b**	64
**4c**	81
**4d**	77
**4e**	65
**4f**	83

**Table 2:** Isolated yields of compounds **6a**, **6b**, and **6f**.

Compounds	Isolated Yield (%)
**6a**	92
**6b**	70
**6f**	60

**Table 3:** Thermal properties for synthesized compounds under N_2_.

Compounds	Td_5_ (°C)	T_max_ (°C)	Residue at 1000 °C (%)
**2**	335	384	23
**4a**	249	403	6
**4b**	219	424	3
**4c**	207	427	27
**4d**	231	426	15
**4e**	236	411	2
**4f**	253	323	8
**6a**	373	384	48
**6b**	238	430	12
**6f**	243	315	39
**8**	308	390	18
**9**	305	350	10

## Data Availability

All data and material described in this work are available in this article or in a Supplementary Materials.

## Supplementary Materials

The following supporting information can be downloaded at: https://www.mdpi.com/article/10.3390/molecules30173583/s1, multinuclear NMR spectra for compounds **2**, **4a**–**4f**, **6a**, **6b**, **6f**, **8**, and **9**; Mass spectra for compounds **2**, **4a**–**4f**, **6a**, **6b**, **6f**, **8**, and **9**. Figure S1: ^1^H NMR spectrum for compound **2**; Figure S2: ^13^C NMR spectrum for compound **2**; Figure S3: ^29^Si NMR spectrum for compound **2**; Figure S4: ^1^H NMR spectrum for compound **4a**; Figure S5: ^13^C NMR spectrum for compound **4a**; Figure S6: ^29^Si NMR spectrum for compound **4a**; Figure S7: ^1^H NMR spectrum for compound **4b**; Figure S8: ^13^C NMR spectrum for compound **4b**; Figure S9: ^29^Si NMR spectrum for compound **4b**; Figure S10: ^1^H NMR spectrum for compound **4c**; Figure S11: ^13^C NMR spectrum for compound **4c**; Figure S12: ^29^Si NMR spectrum for compound **4c**; Figure S13: ^1^H NMR spectrum for compound **4d**; Figure S14: ^13^C NMR spectrum for compound **4d**; Figure S15: ^29^Si NMR spectrum for compound **4d**; Figure S16: ^1^H NMR spectrum for compound **4e**; Figure S17: ^13^C NMR spectrum for compound **4e**; Figure S18: ^29^Si NMR spectrum for compound **4e**; Figure S19: ^19^F NMR spectrum for compound **4e**; Figure S20: ^1^H NMR spectrum for compound **4f**; Figure S21: ^13^C NMR spectrum for compound **4f**; Figure S22: ^29^Si NMR spectrum for compound **4f**; Figure S23: ^1^H NMR spectrum for compound **6a**; Figure S24: ^13^C NMR spectrum for compound **6a**; Figure S25: ^29^Si NMR spectrum for compound **6a**; Figure S26: ^1^H NMR spectrum for compound **6b**; Figure S27: ^13^C NMR spectrum for compound **6b**; Figure S28: ^29^Si NMR spectrum for compound **6b**; Figure S29: ^1^H NMR spectrum for compound **6f**; Figure S30: ^13^C NMR spectrum for compound **6f**; Figure S31: ^29^Si NMR spectrum for compound **6f**; Figure S32: ^1^H NMR spectrum for compound **8**; Figure S33: ^13^C NMR spectrum for compound **8**; Figure S34: ^29^Si NMR spectrum for compound **8**; Figure S35: ^1^H NMR spectrum for compound **9**; Figure S36: ^13^C NMR spectrum for compound **9**; Figure S37: ^29^Si NMR spectrum for compound **9**; Figure S38: MALDI-TOF analysis for compound **2**; Figure S39: MALDI-TOF analysis for compound **4a**; Figure S40: MALDI-TOF analysis for compound **4b**; Figure S41: MALDI-TOF analysis for compound **4c**; Figure S42: MALDI-TOF analysis for compound **4d**; Figure S43: MALDI-TOF analysis for compound **4e**; Figure S44: HRMS analysis for compound **4f**; Figure S45: MALDI-TOF analysis for compound **6a**; Figure S46: MALDI-TOF analysis for compound **6b**; Figure S47: MALDI-TOF analysis for compound **8**; Figure S48: MALDI-TOF analysis for compound **9**; Figure S49: Thermogravimetric graphs for all-cis-T_4_ compounds; Figure S50: Thermogravimetric graphs for T_8_ compounds; Figure S51: Thermogravimetric graphs for laddersiloxanes.
