# Supplementary material for: Unlocking New Potential in the Functionalization of Chlorinated Silsesquioxanes: A Rapid and Chemoselective Thiolation Method
Source: Molecules. 2025 Sep 2;30(17):3583. doi: 10.3390/molecules30173583 (PMC12430186; doi:10.3390/molecules30173583)
Supplement: Supplementary file 1 [file molecules-30-03583-s001.zip › molecules-3817186-supplementary.pdf]

Unlocking New Potential in the Functionalization of Chlorinated Silsesquioxanes: A Rapid and Chemoselective Thiolation Method

Niyaz Yagafarov[1], Yujia Liu[1*], Naoto Adachi[1], Nobuhiro Takeda[1], Masafumi Unno[1*], and Armelle Ouali[2*]

[1] Department of Chemistry and Chemical Biology, Graduate School of Science and Technology, Gunma University, 1-5-1 Tenjin-cho, Kiryu 376-8515, Japan; niiaz.iagafarov@gmail.com (N.Y.); ntakeda@gunma-u.ac.jp (N.T.).
[2] ICGM, Univ Montpellier, CNRS, ENSCM (Institut Charles Gerhardt Montpellier, Université de Montpellier, Center National de la Recherche Scientifique, École Nationale Supérieure de Chimie de Montpellier), 1919 Route de Mende, Cedex 05, 34293 Montpellier, France.
* Correspondence: yliu@gunma-u.ac.jp (Y.L.) ; unno@gunma-u.ac.jp (M.U.) ;armelle.ouali@enscm.fr (A.O.) ; Tel. : +81-27-730-1234 (Y.L., M.U.) ; +33-44-879-2010 (A.O.).

Supporting information

Table of contents **Pages**

1. Multinuclear NMR spectra for compounds **2-9** 2
2. MALDI-TOF mass spectra for compounds **2-9** 21
3. Thermogravimetric graphs for compounds **2-9** 26

1. Multinuclear NMR spectra for compounds 2, 4a-4f, 6a, 6b, 6f, 8 and 9

Compound 2

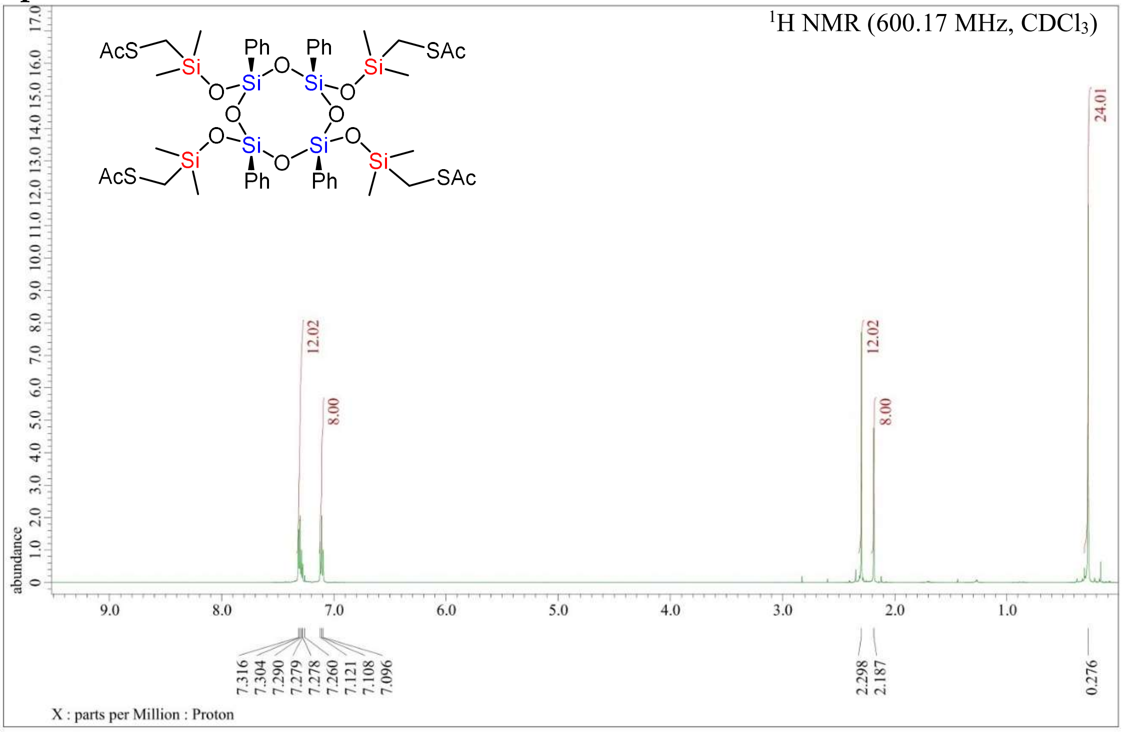

Figure S1: ^{1}H NMR spectrum for compound **2**

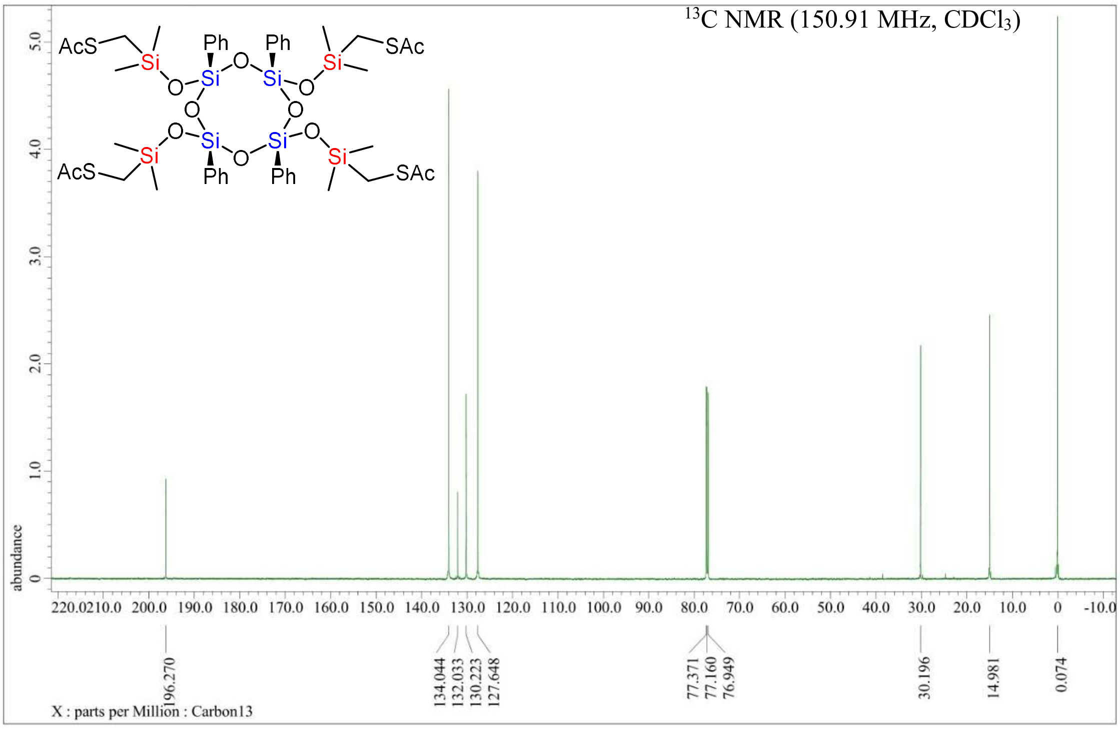

Figure S2: ^{13}C NMR spectrum for compound **2**

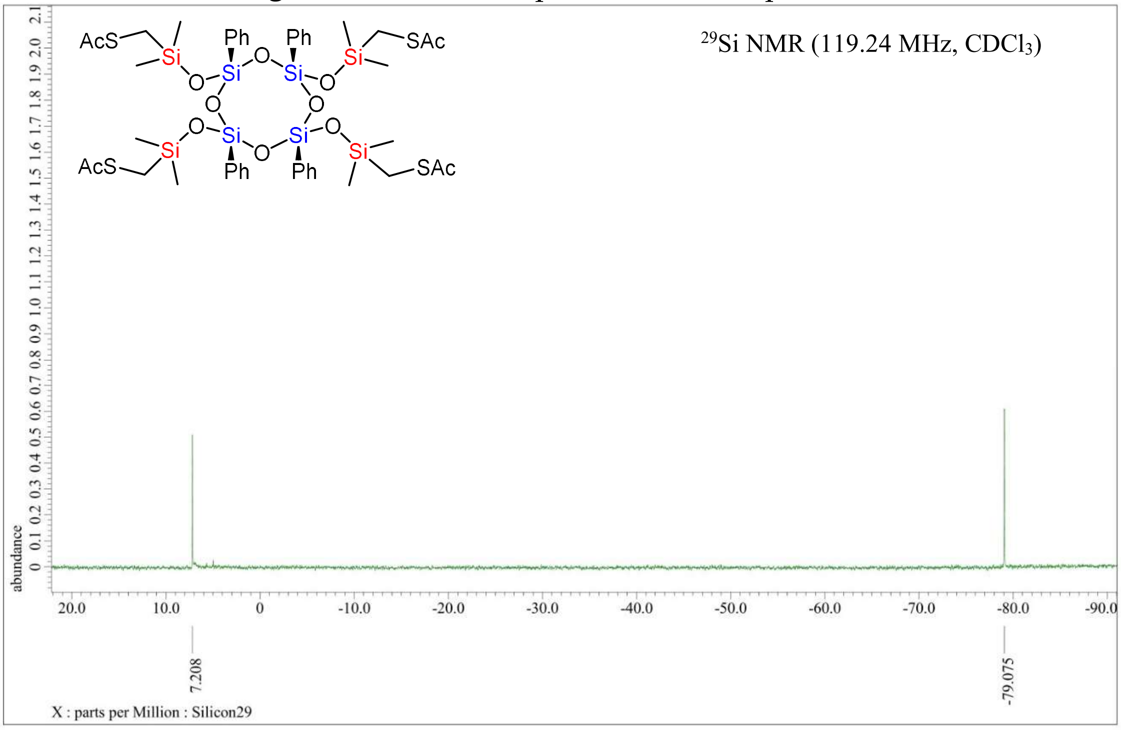

Figure S3: ^{29}Si NMR spectrum for compound **2**

Compound 4a

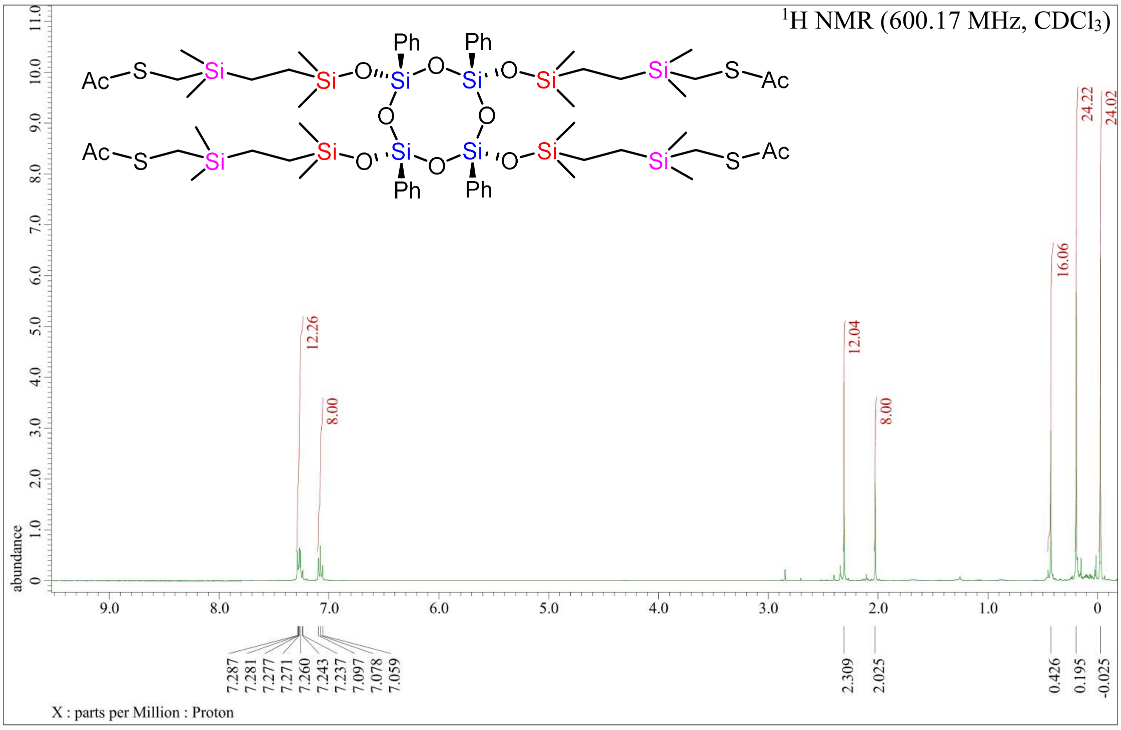

Figure S4: ^{1}H NMR spectrum for compound **4a**

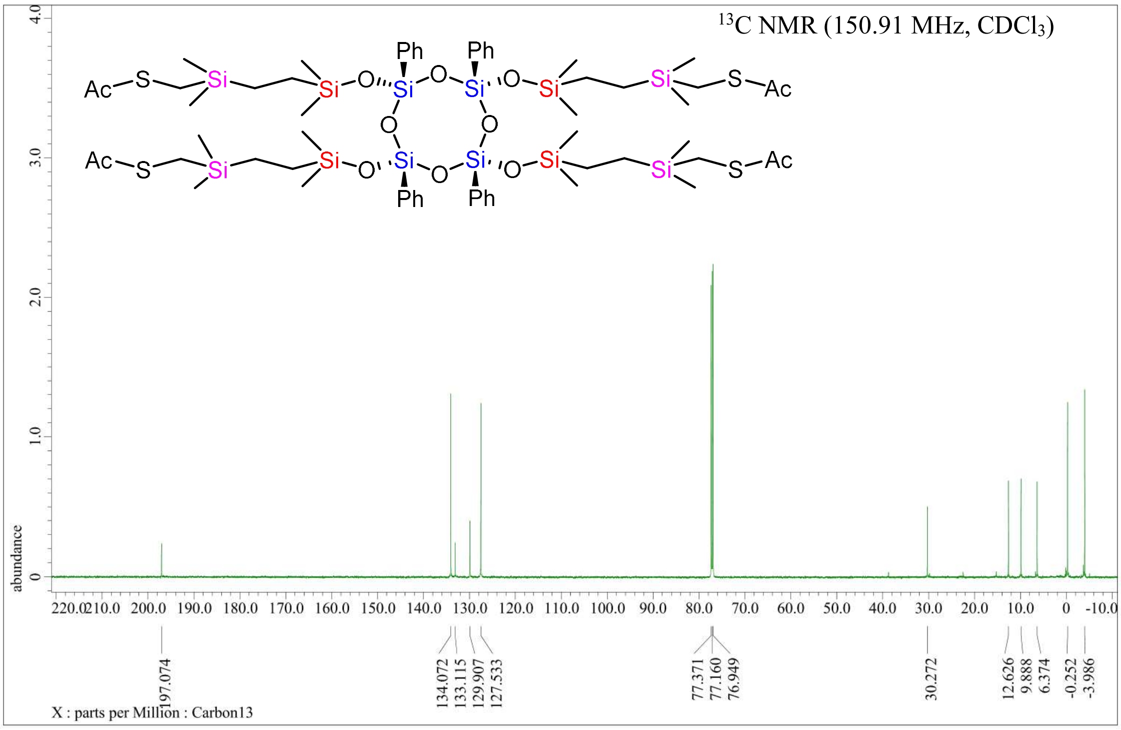

Figure S5: ^{13}C NMR spectrum for compound **4a**

^{29}Si NMR (119.24 MHz, $CDCl_3$)

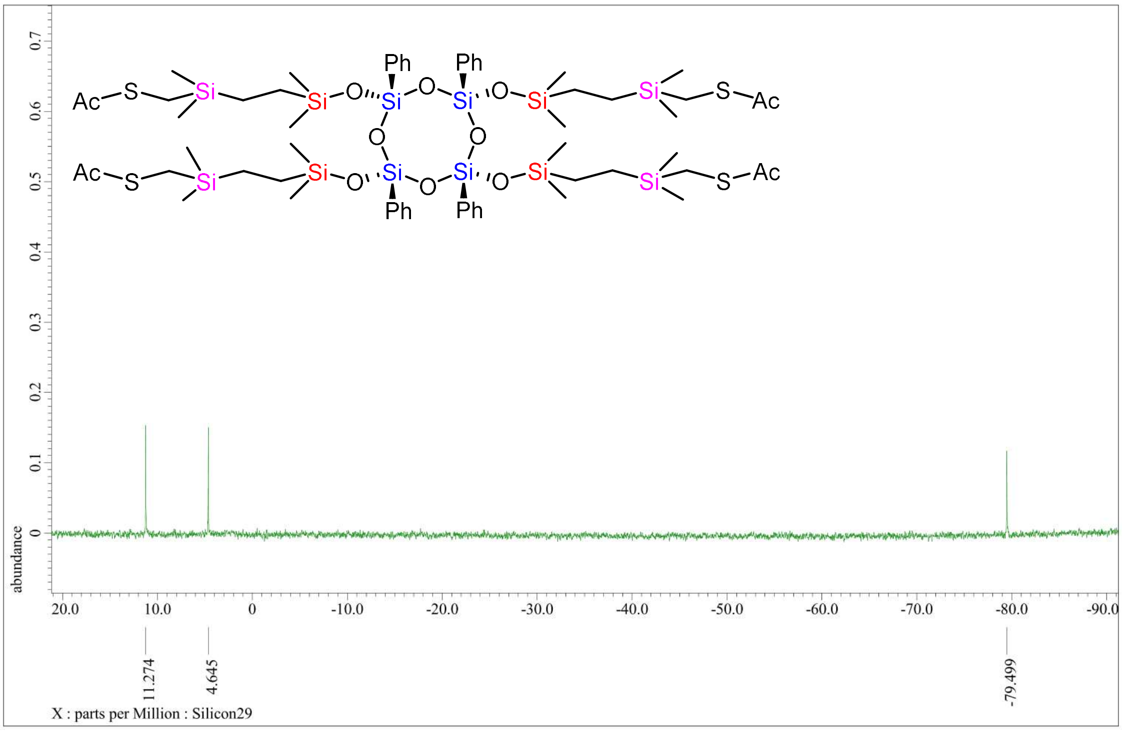

Figure S6: ^{29}Si NMR spectrum for compound **4a**

Compound 4b

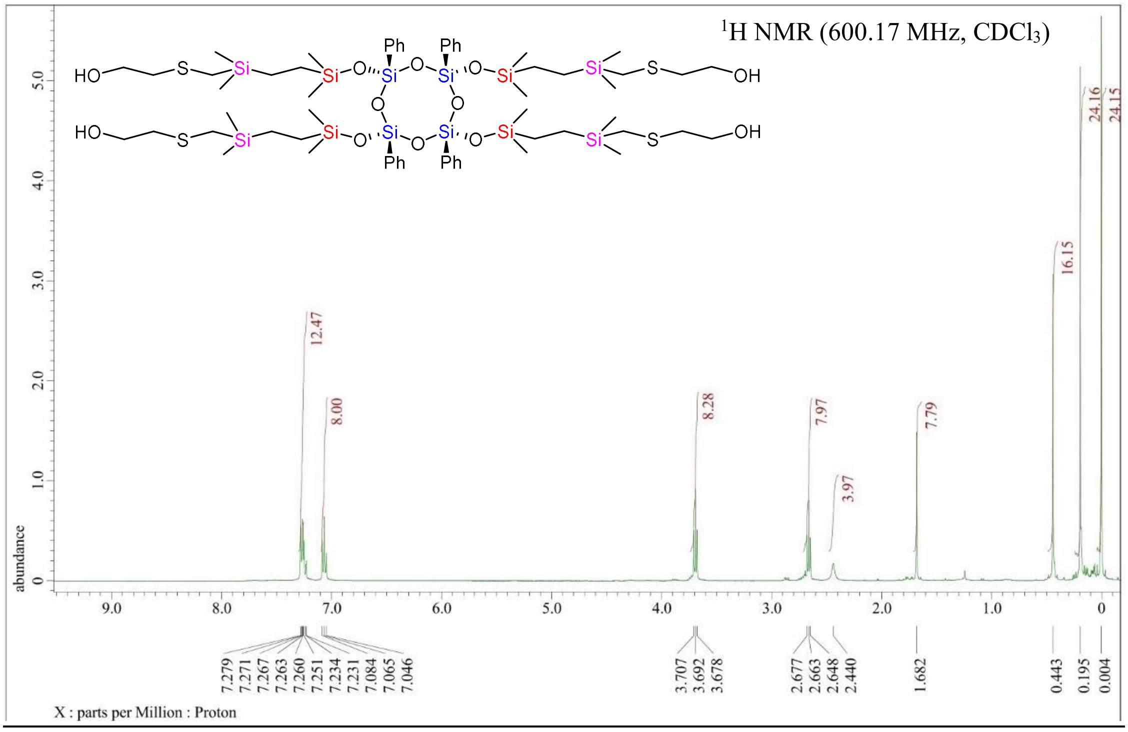

Figure S7: ^{1}H NMR spectrum for compound **4b**

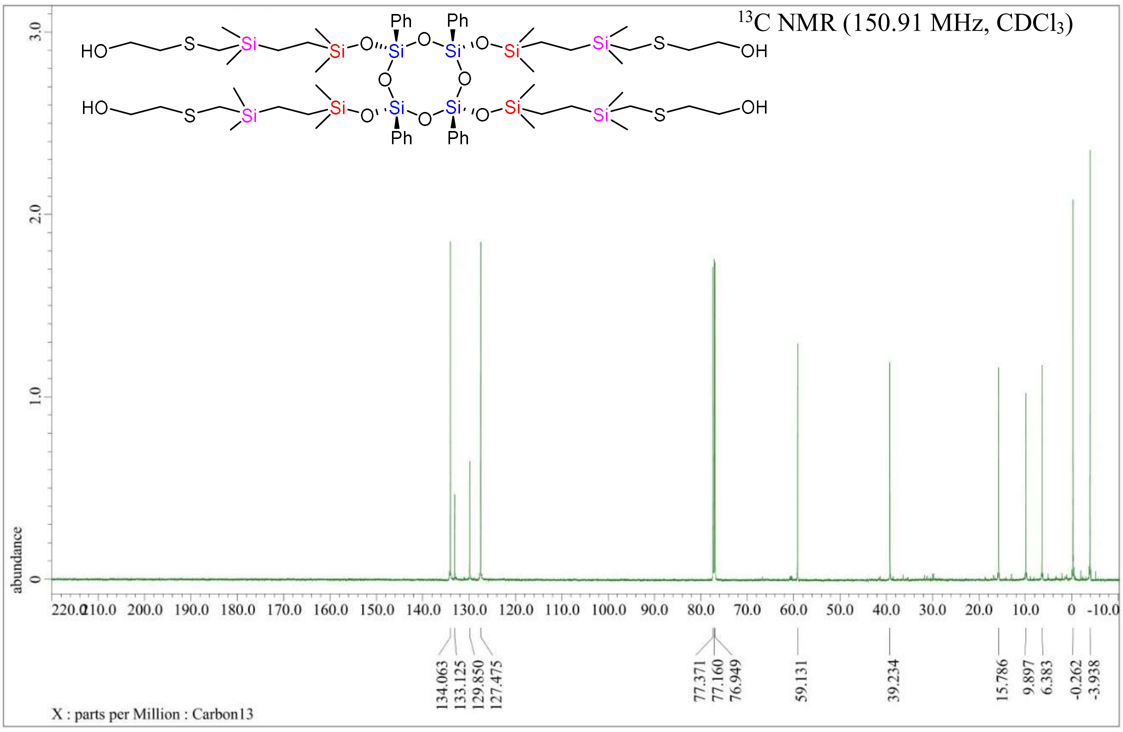

Figure S8: ^{13}C NMR spectrum for compound **4b**

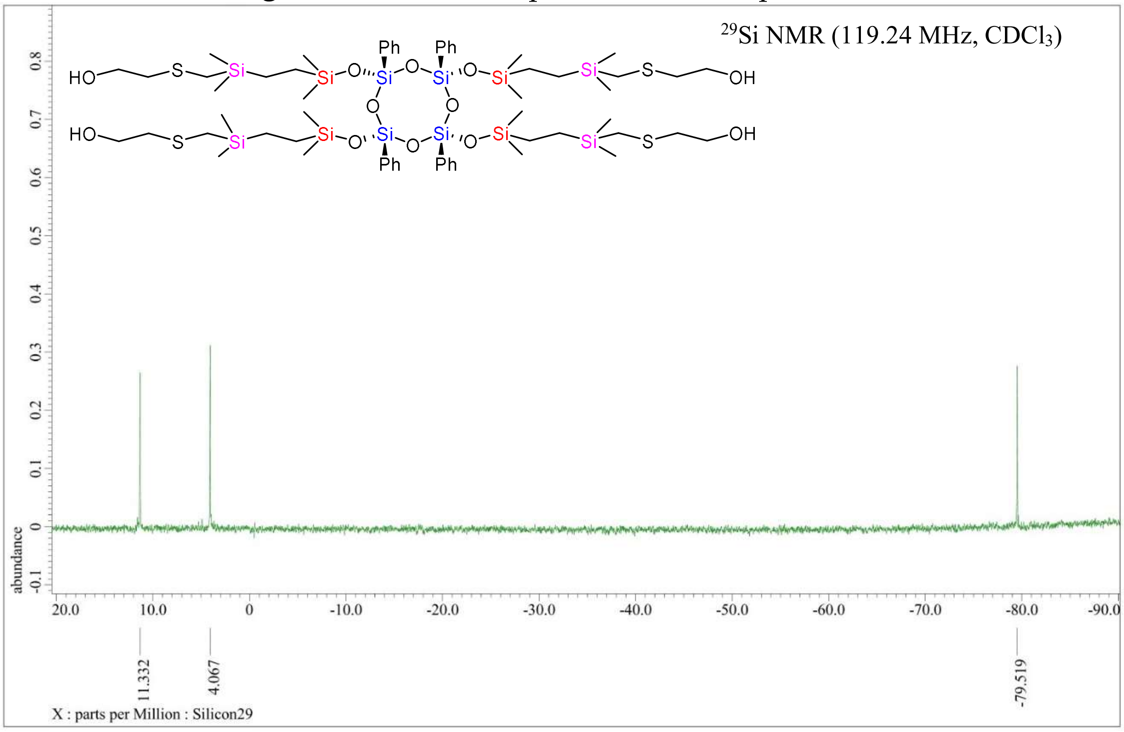

Figure S9: ^{29}Si NMR spectrum for compound **4b**

Compound 4c

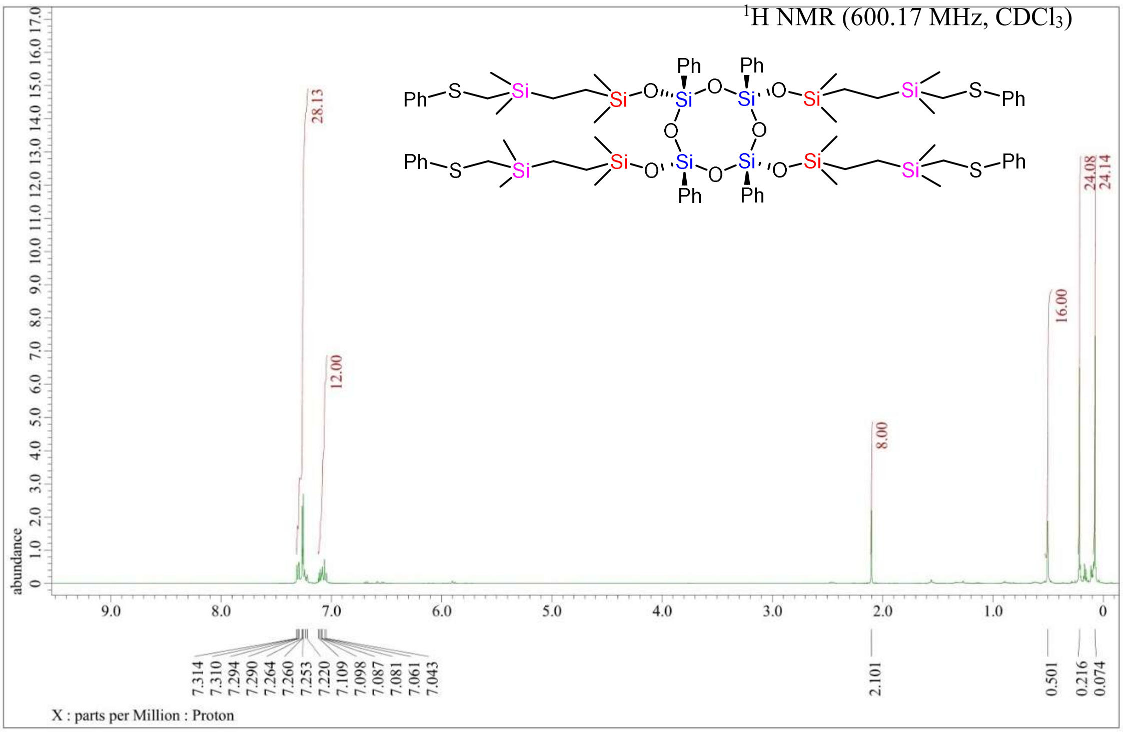

Figure S10: ^{1}H NMR spectrum for compound **4c**

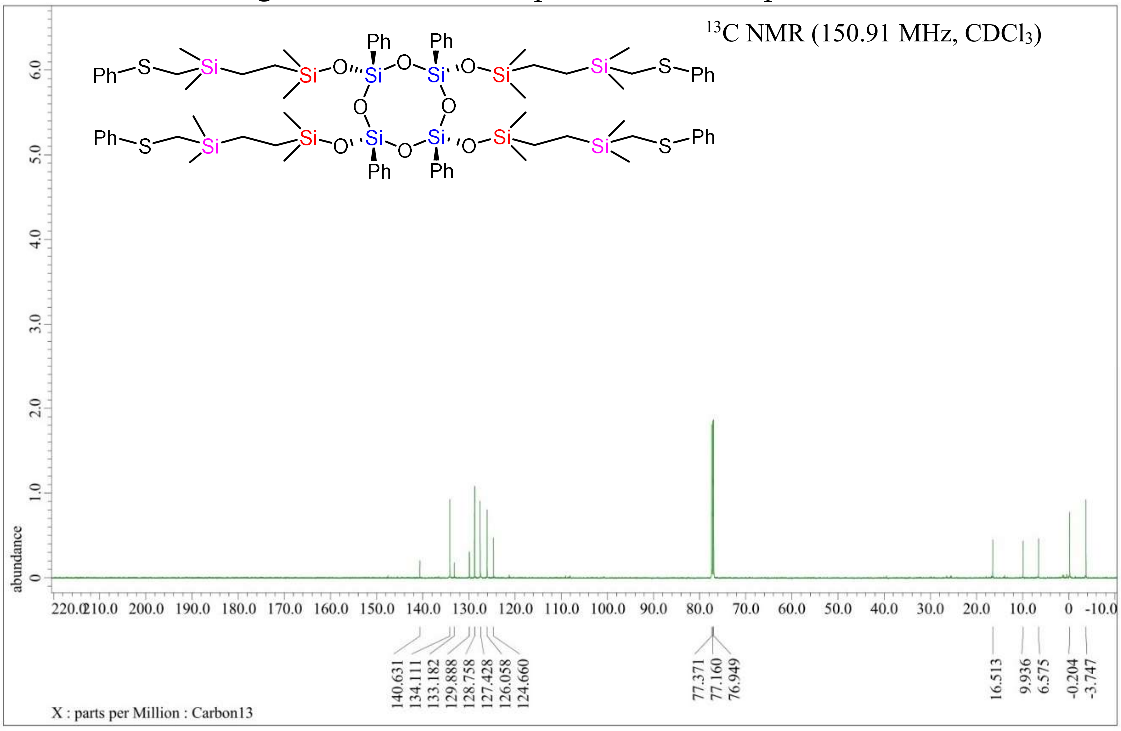

Figure S11: ^{13}C NMR spectrum for compound **4c**

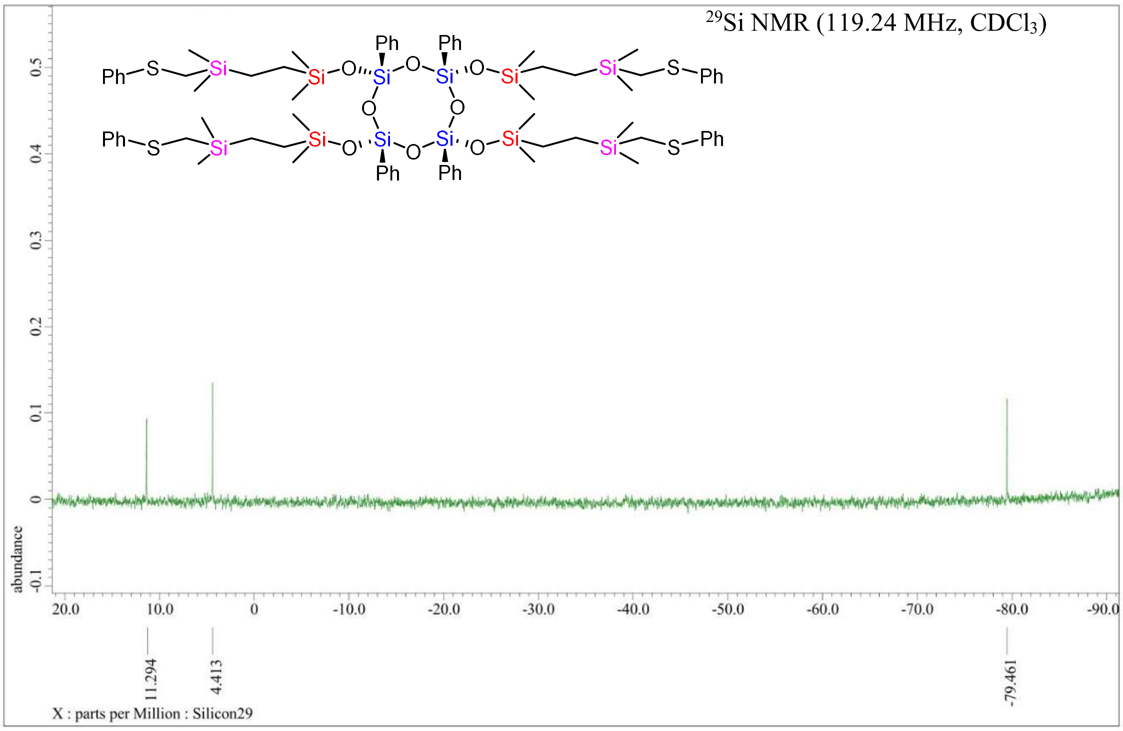

Figure S12: ^{29}Si NMR spectrum for compound **4c**

Compound 4d

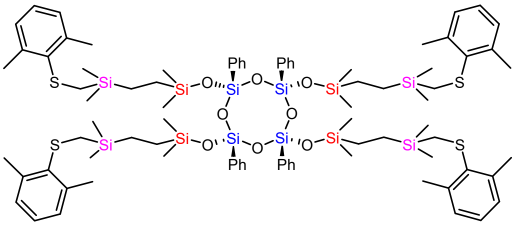

^{1}H NMR (600.17 MHz, $CDCl_3$)

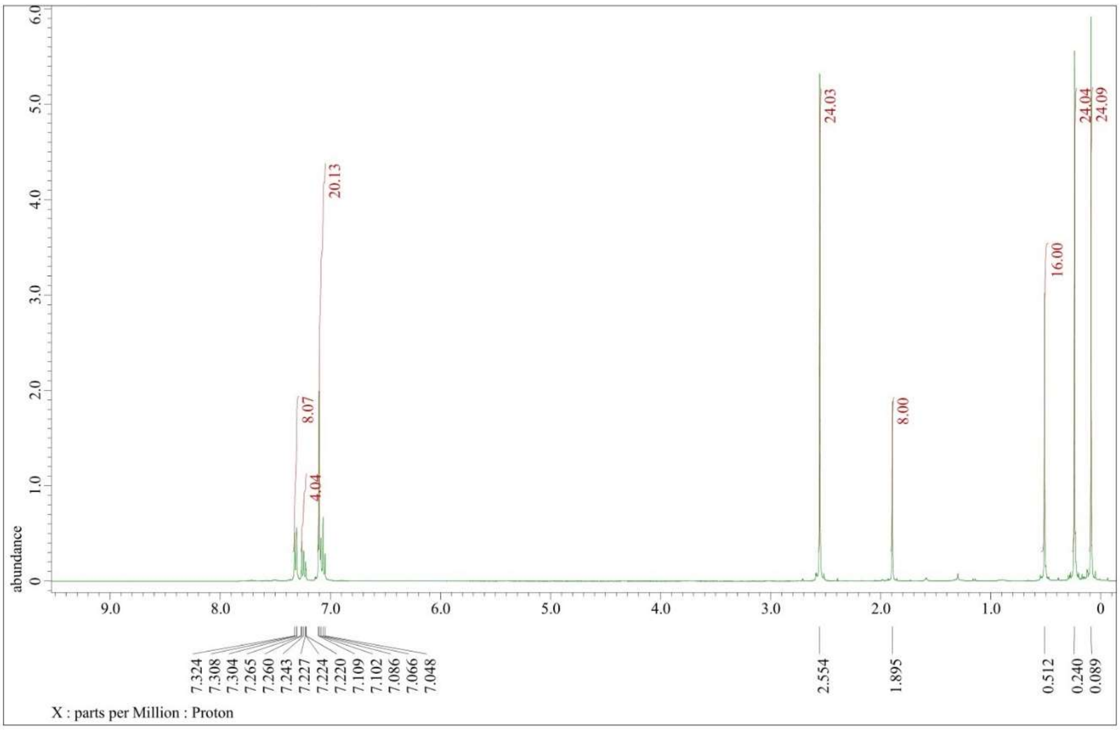

Figure S13: ^{1}H NMR spectrum for compound **4d**

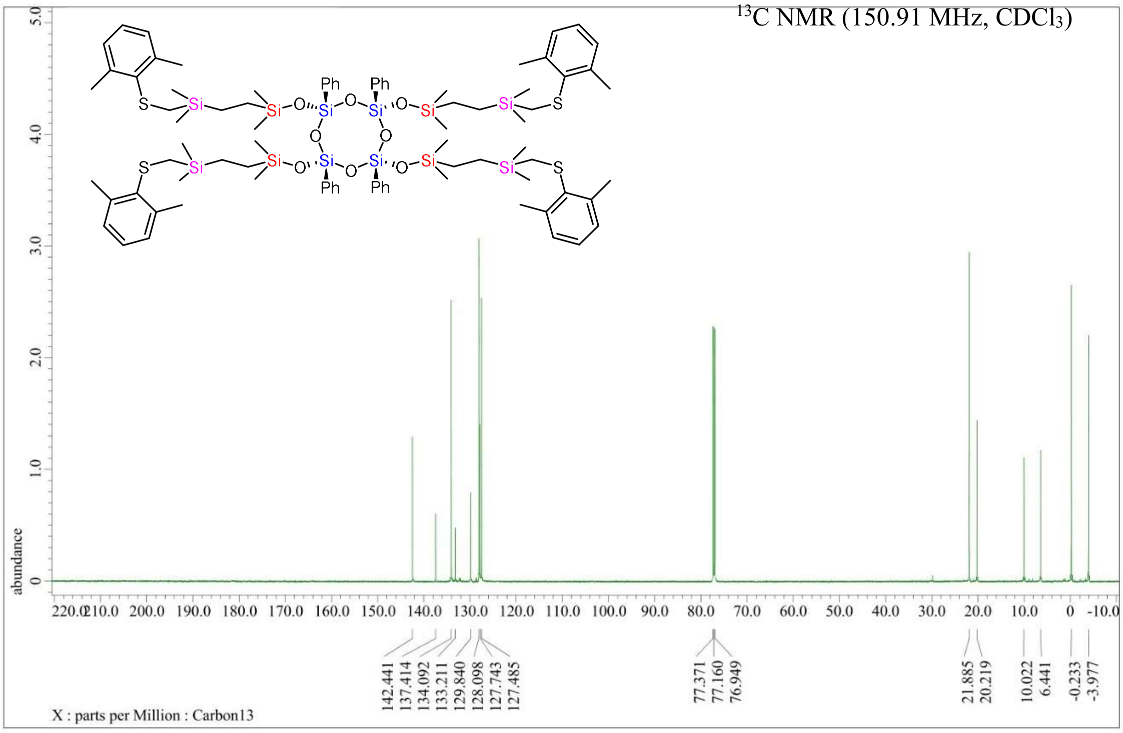

Figure S14: ^{13}C NMR spectrum for compound **4d**

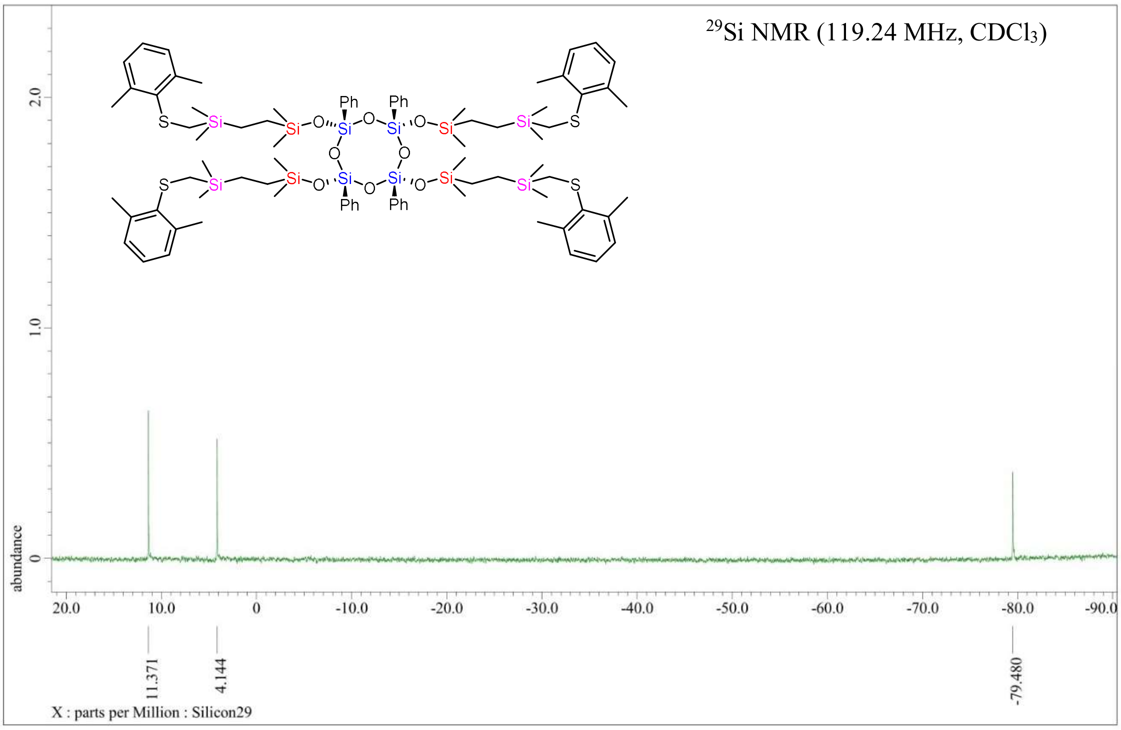

Figure S15: ^{29}Si NMR spectrum for compound **4d**

<u>Compound 4e</u>

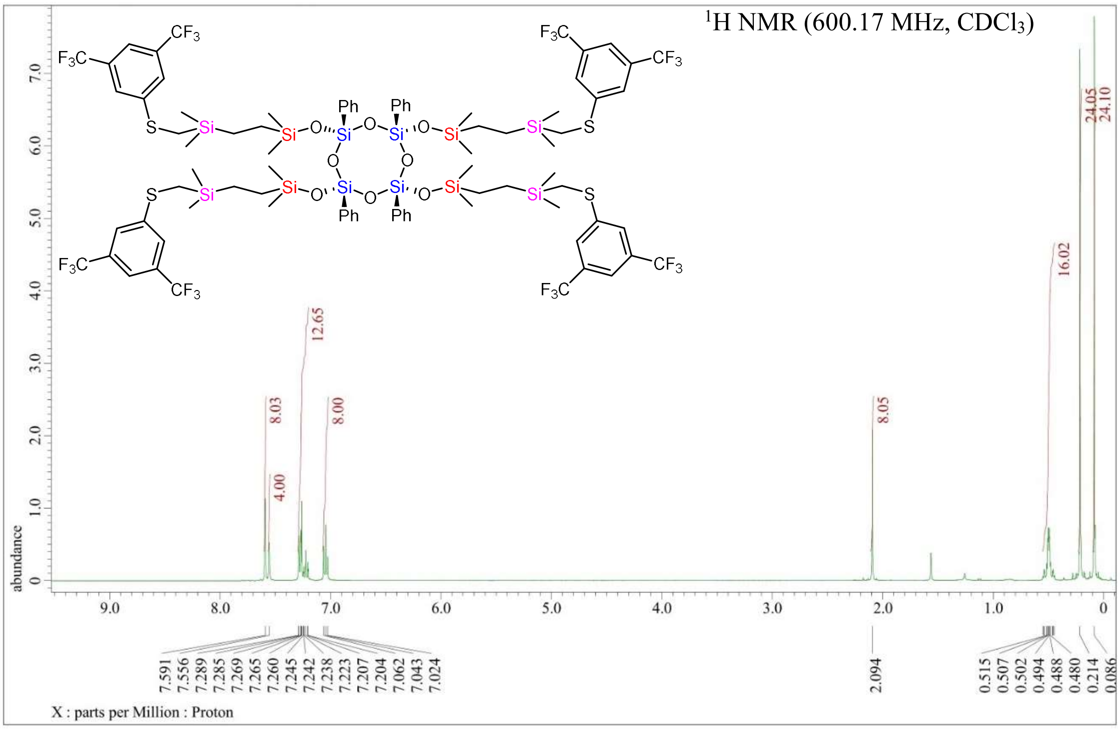

Figure S16: ^{1}H NMR spectrum for compound **4e**

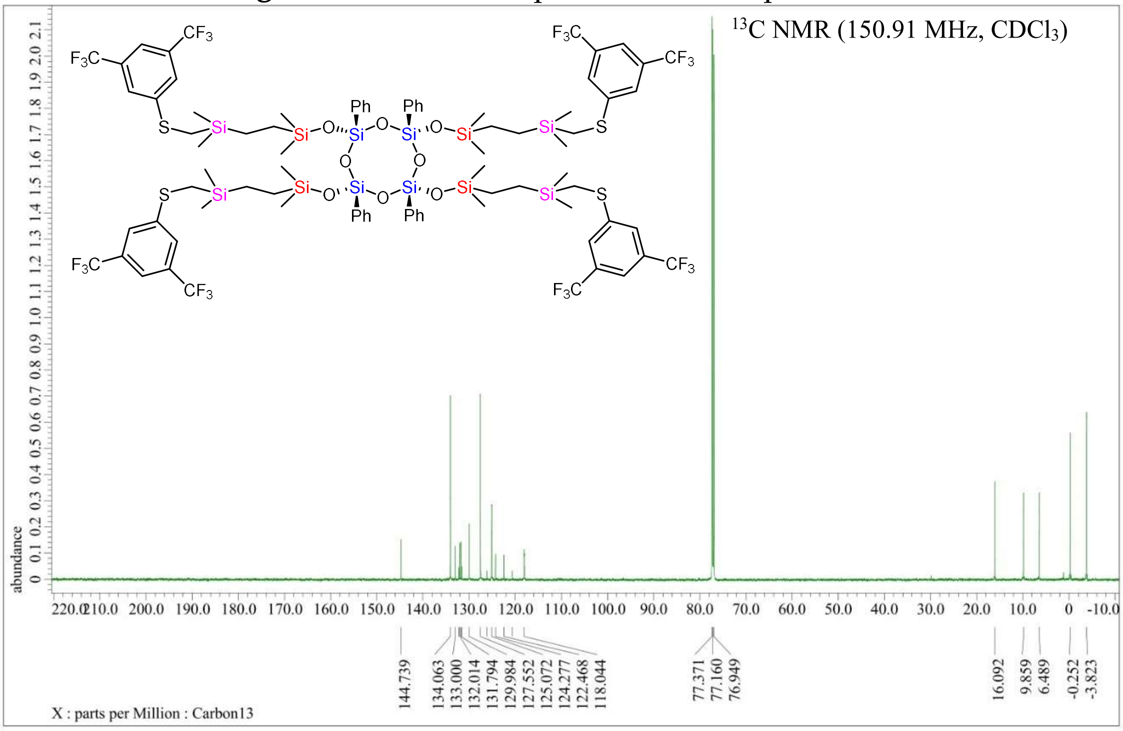

Figure S17: ^{13}C NMR spectrum for compound **4e**

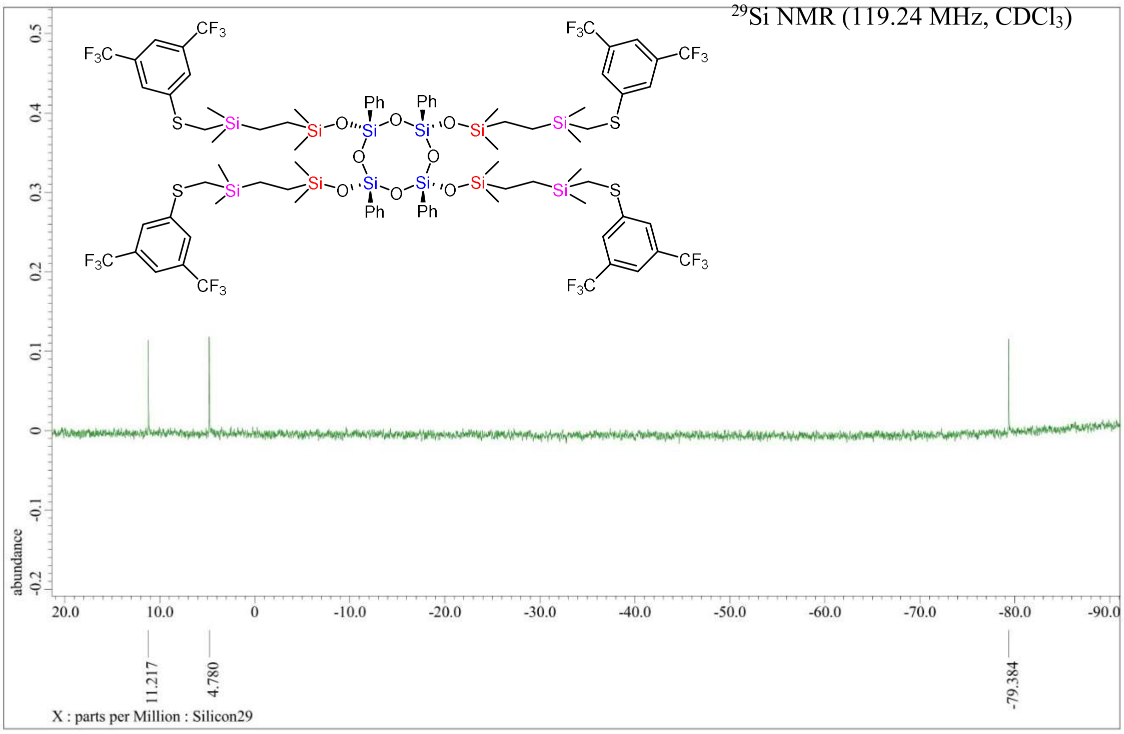

Figure S18: ^{29}Si NMR spectrum for compound **4e**

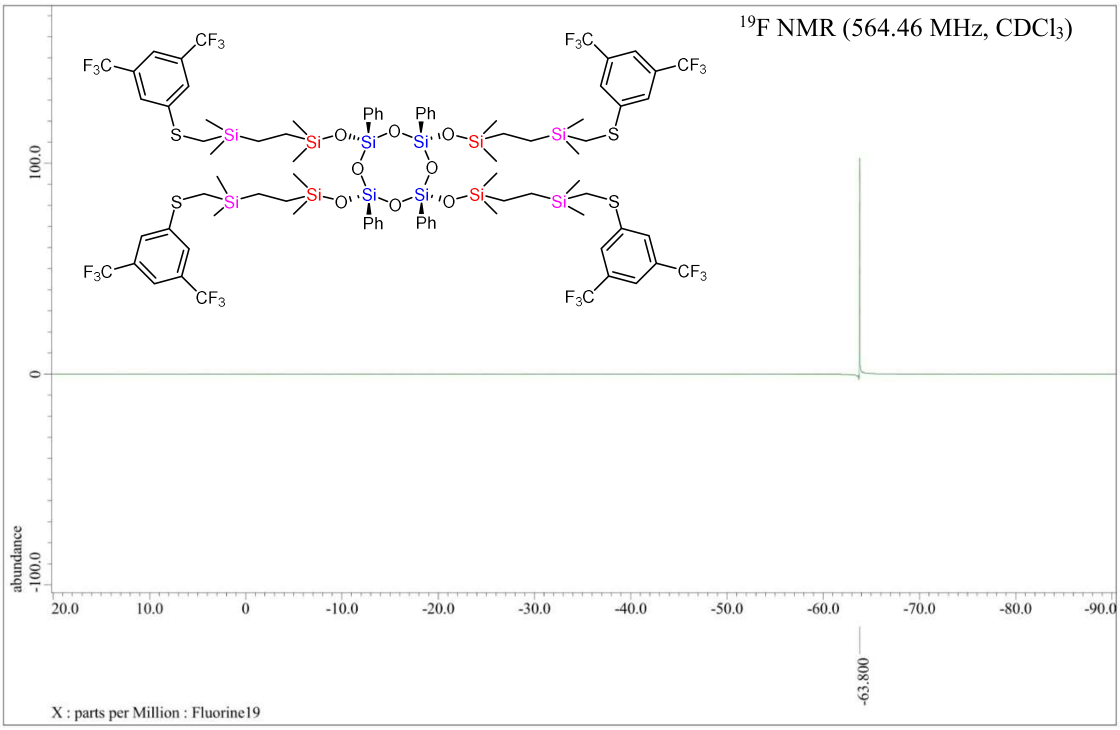

Figure S19: ^{19}F NMR spectrum for compound **4e**

<u>Compound 4f</u>

^{1}H NMR (600.17 MHz, $CDCl_3$)

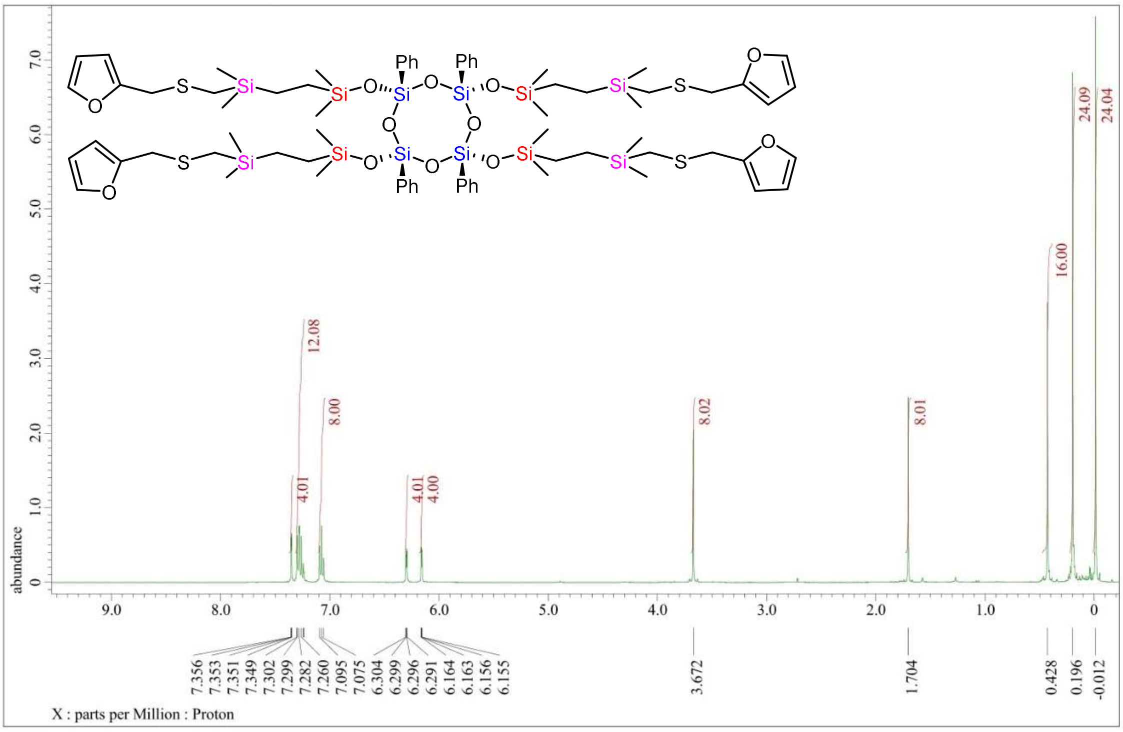

Figure S20: ^{1}H NMR spectrum for compound **4f**

^{13}C NMR (150.91 MHz, $CDCl_3$)

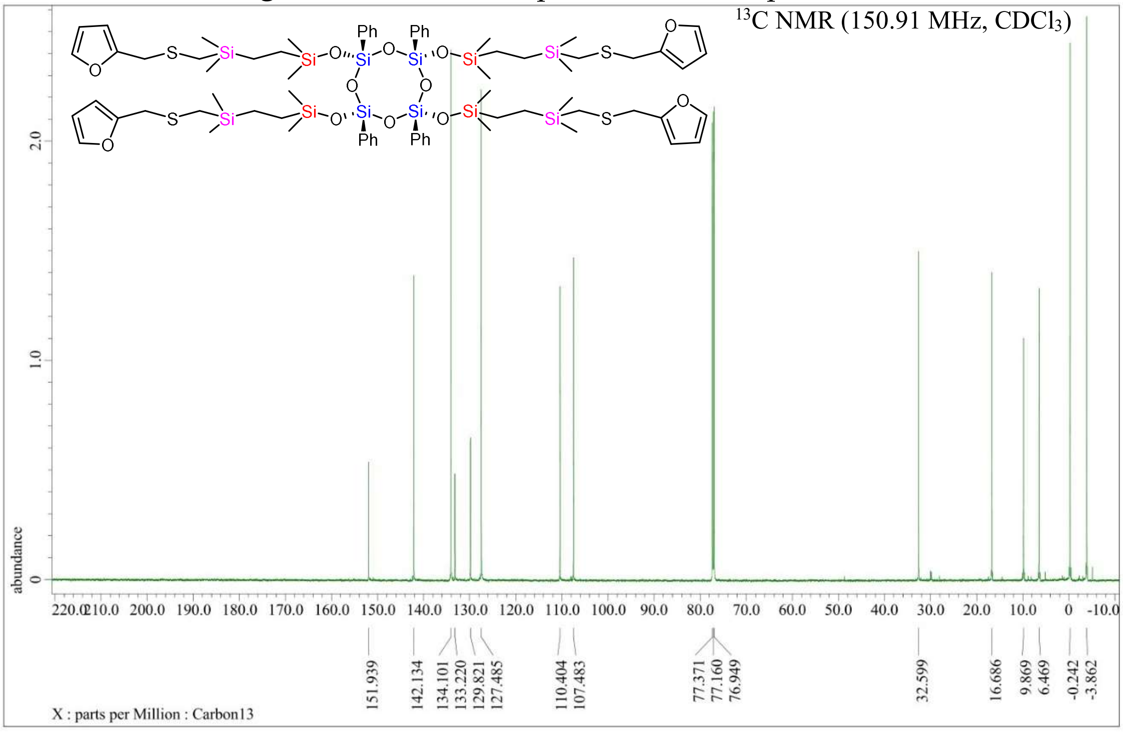

Figure S21: ^{13}C NMR spectrum for compound **4f**

^{29}Si NMR (119.24 MHz, $CDCl_3$)

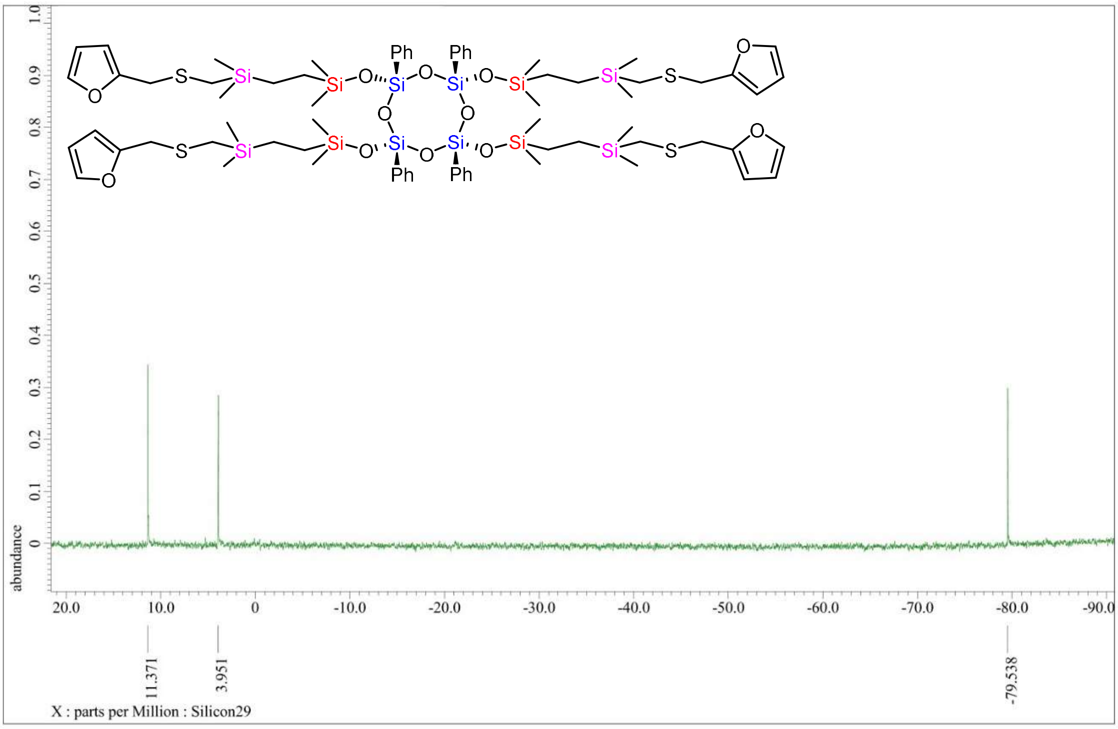

Figure S22: ^{29}Si NMR spectrum for compound **4f**

Compound 6a

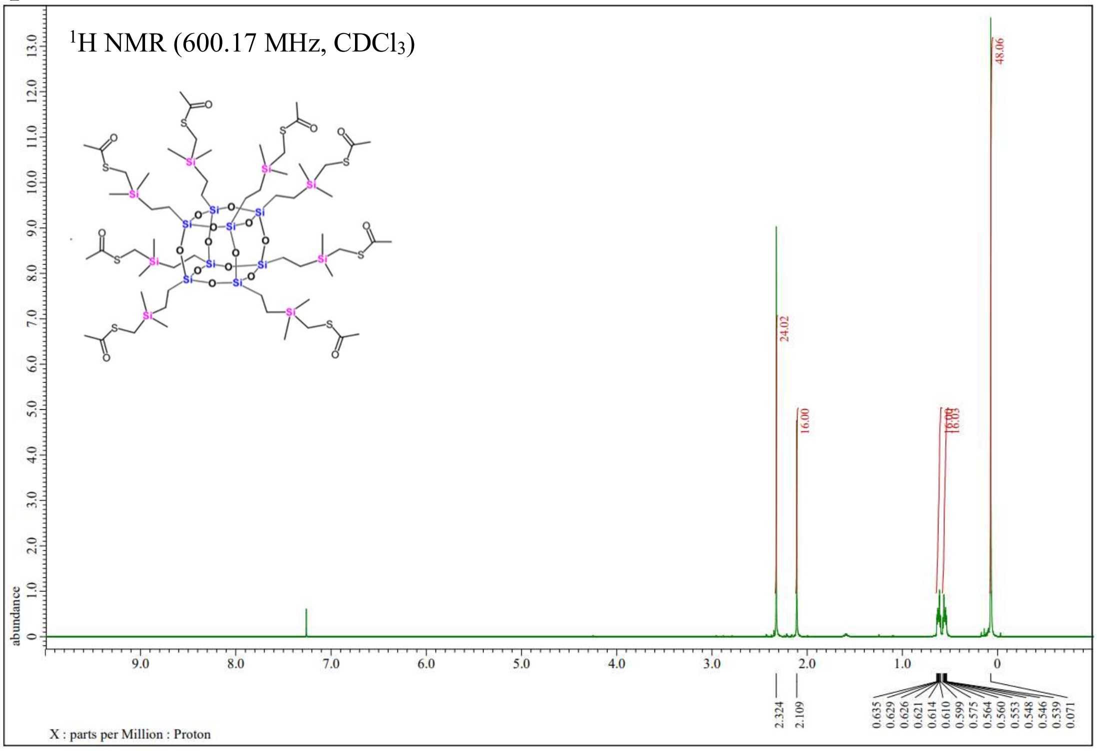

Figure S23: ^{1}H NMR spectrum for compound **6a**

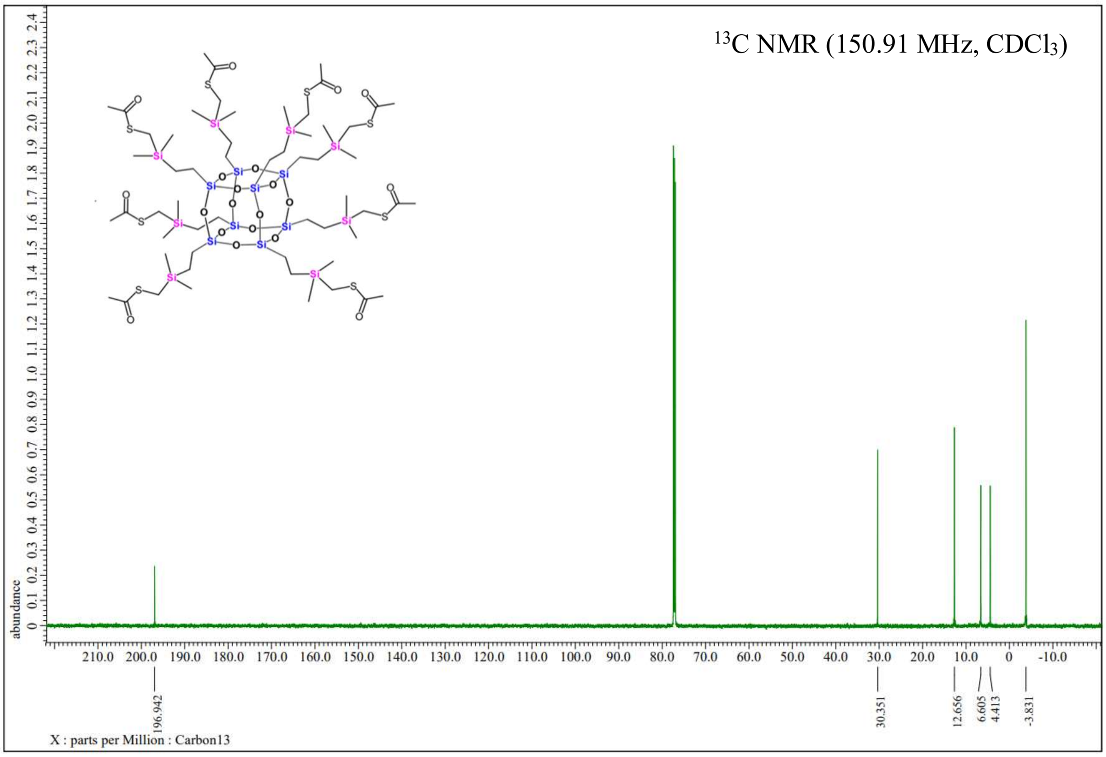

Figure S24: ^{13}C NMR spectrum for compound **6a**

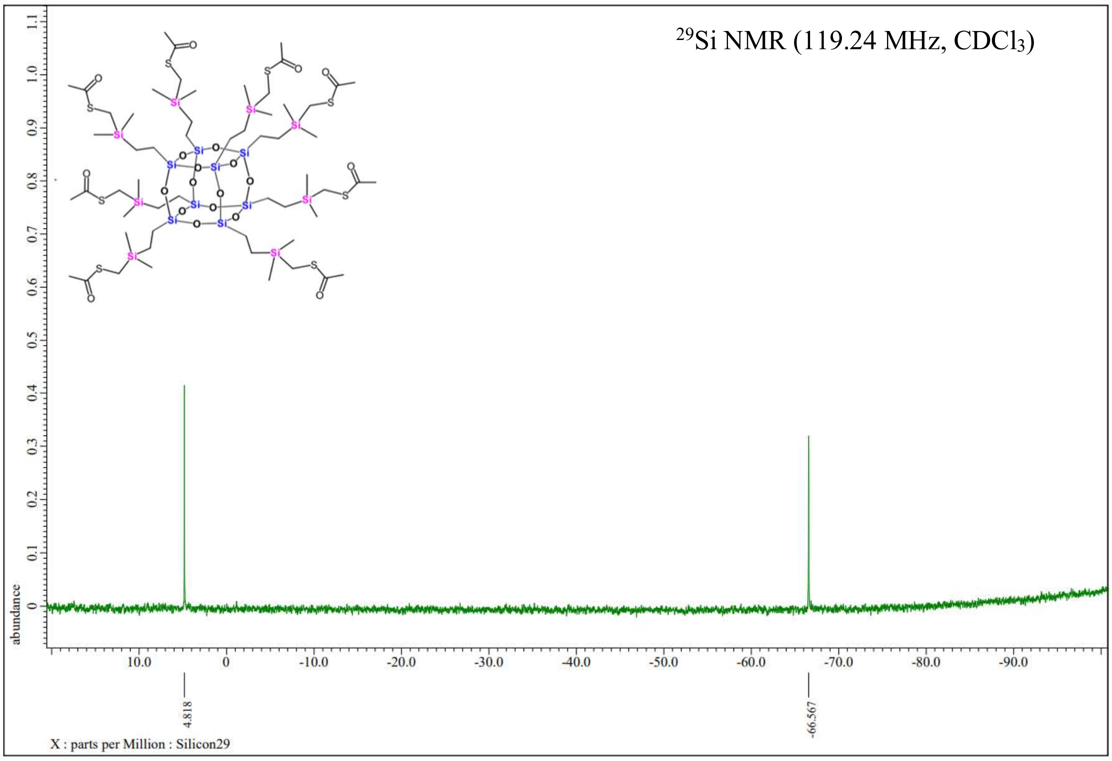

Figure S25: ^{29}Si NMR spectrum for compound **6a**

Compound 6b

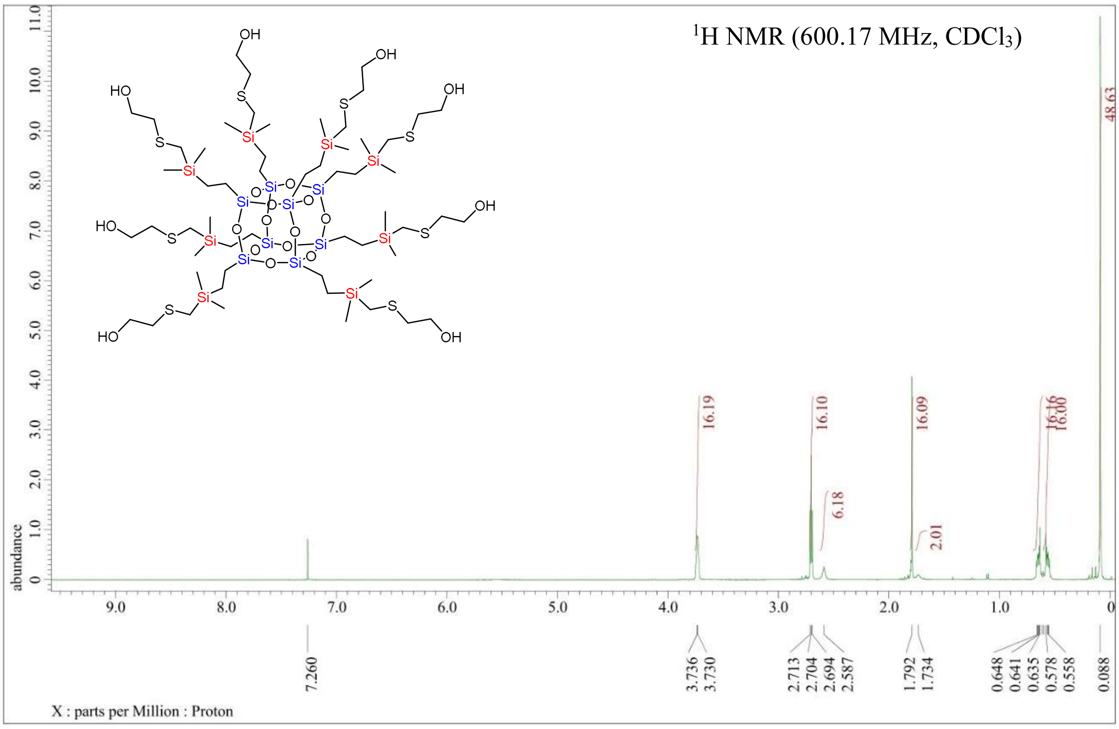

Figure S26: ^{1}H NMR spectrum for compound **6b**

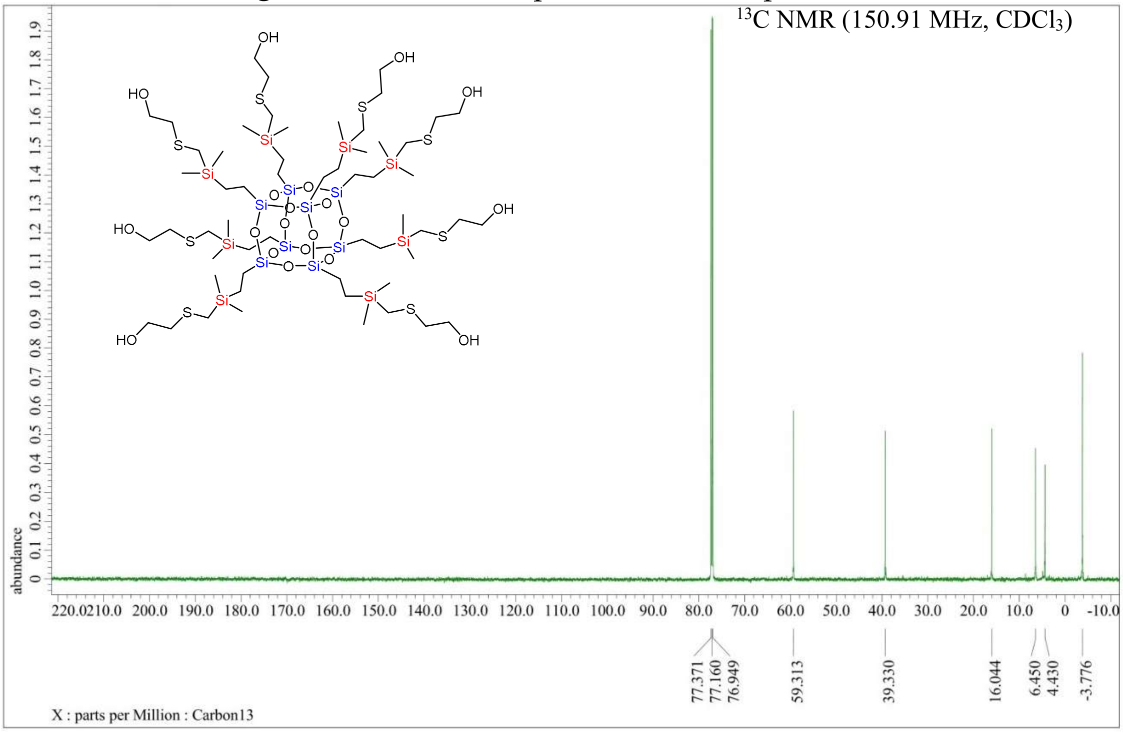

Figure S27: ^{13}C NMR spectrum for compound **6b**

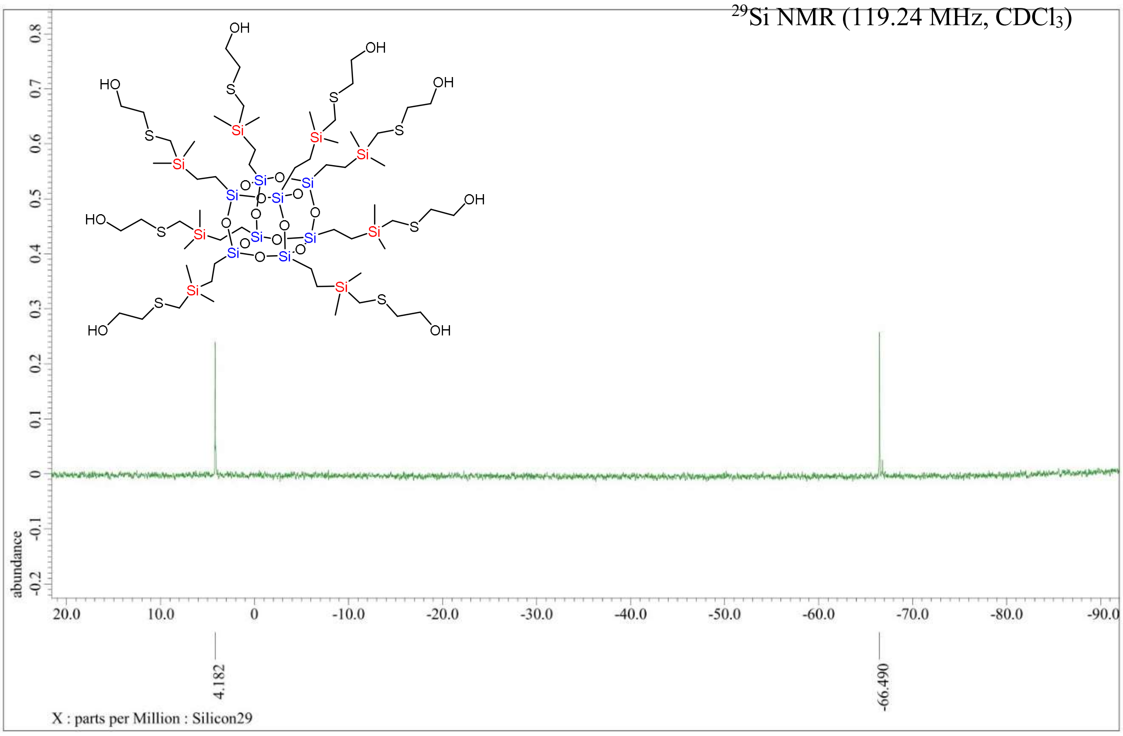

Figure S28: ^{29}Si NMR spectrum for compound **6b**

Compound 6f

^{1}H NMR (600.17 MHz, $CDCl_3$)

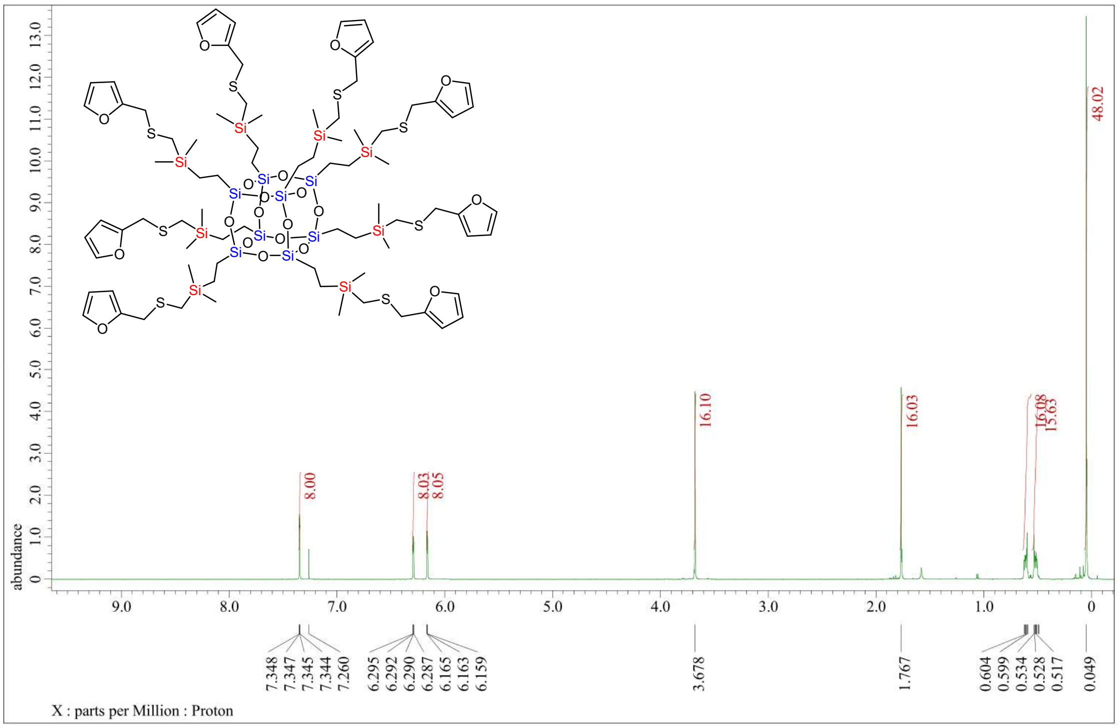

Figure S29: ^{1}H NMR spectrum for compound **6f**

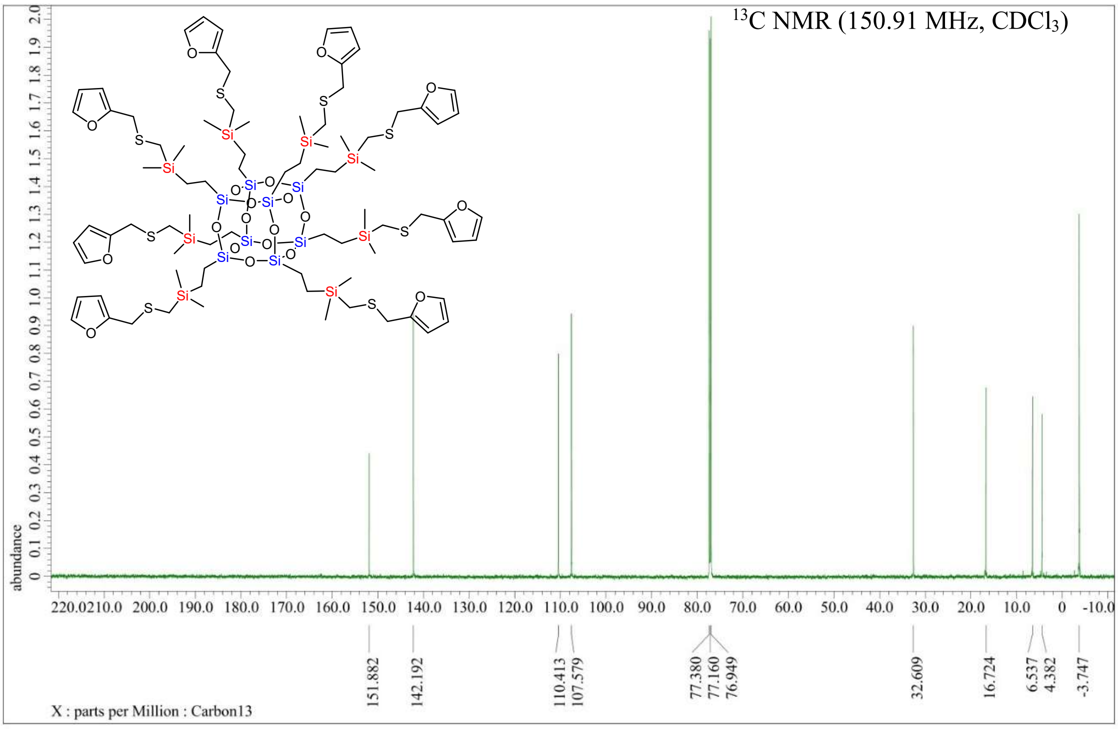

Figure S30: ^{13}C NMR spectrum for compound **6f**

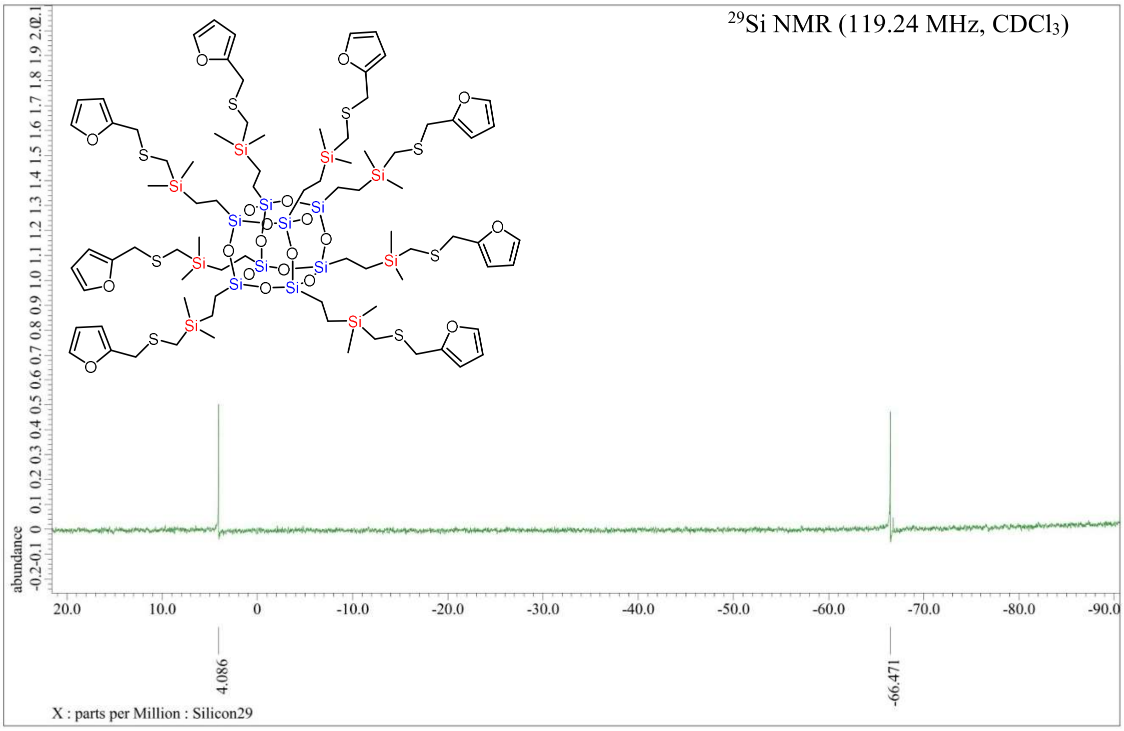

Figure S31: ^{29}Si NMR spectrum for compound **6f**

Compound 8

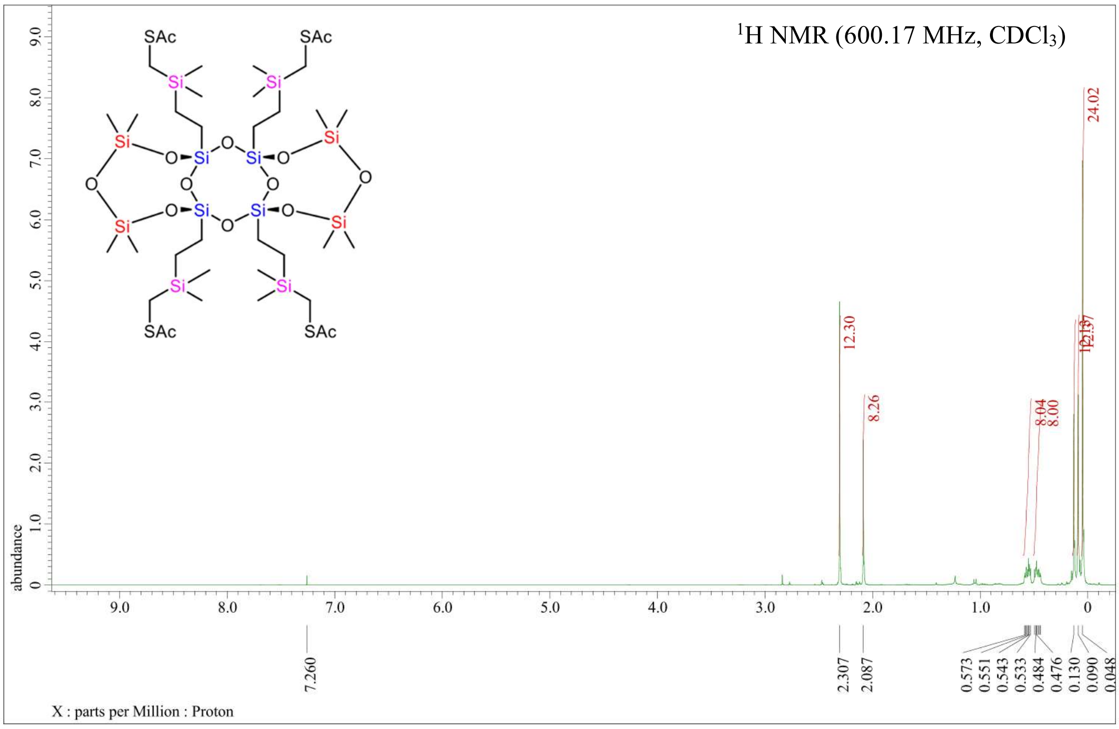

Figure S32: ¹H NMR spectrum for compound **8**

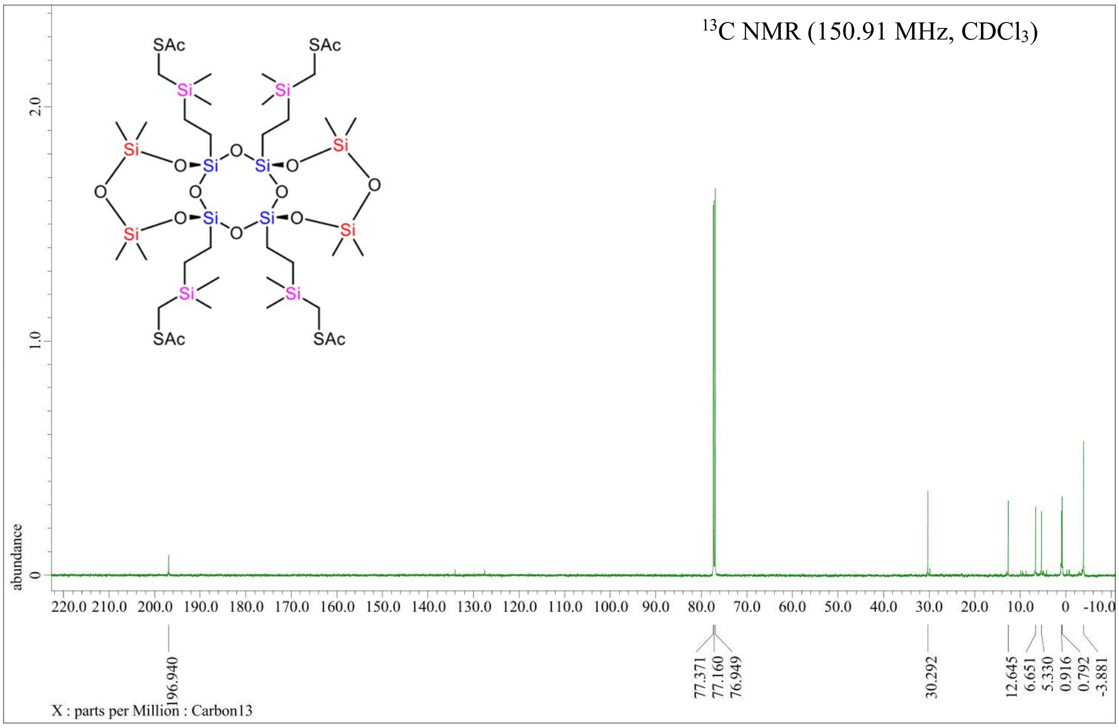

Figure S33: ^{13}C NMR spectrum for compound **8**

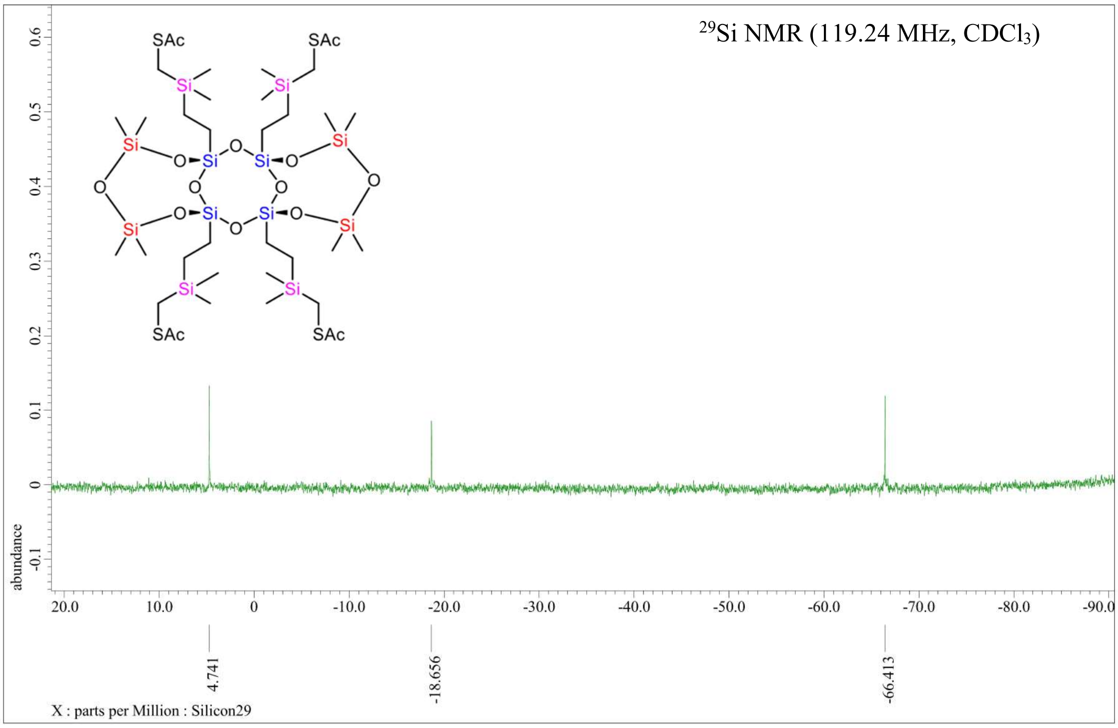

Figure S34: ^{29}Si NMR spectrum for compound **8**

Compound 9

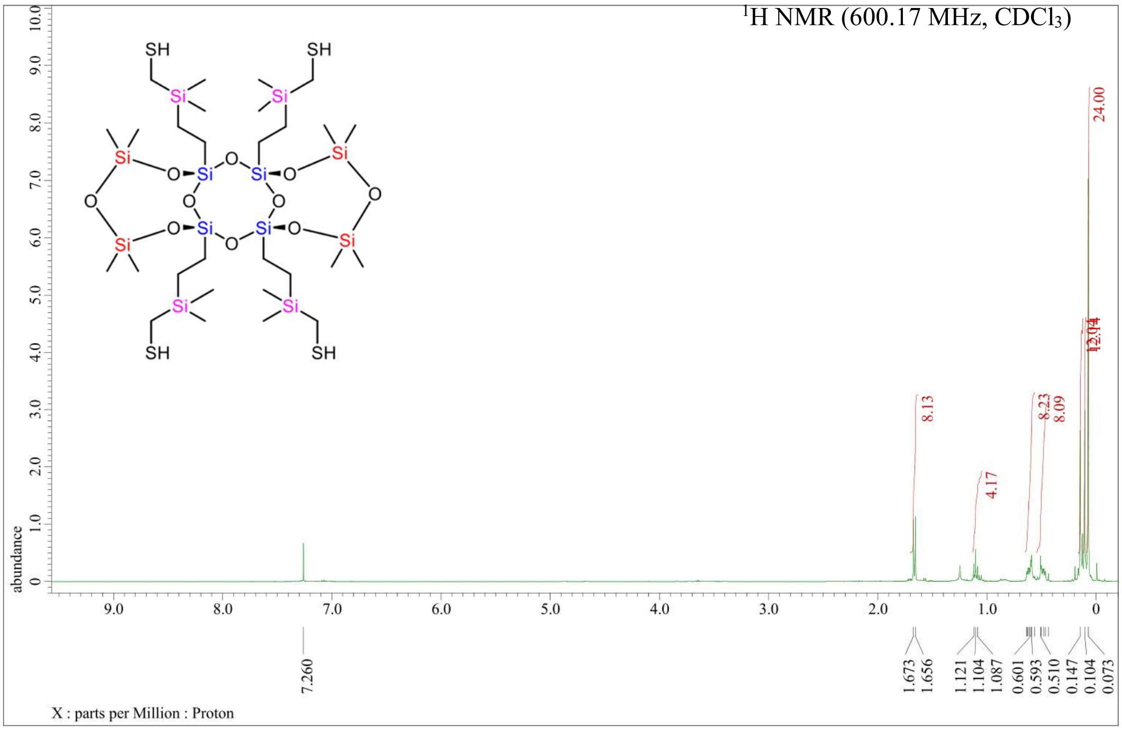

Figure S35: ^{1}H NMR spectrum for compound **9**

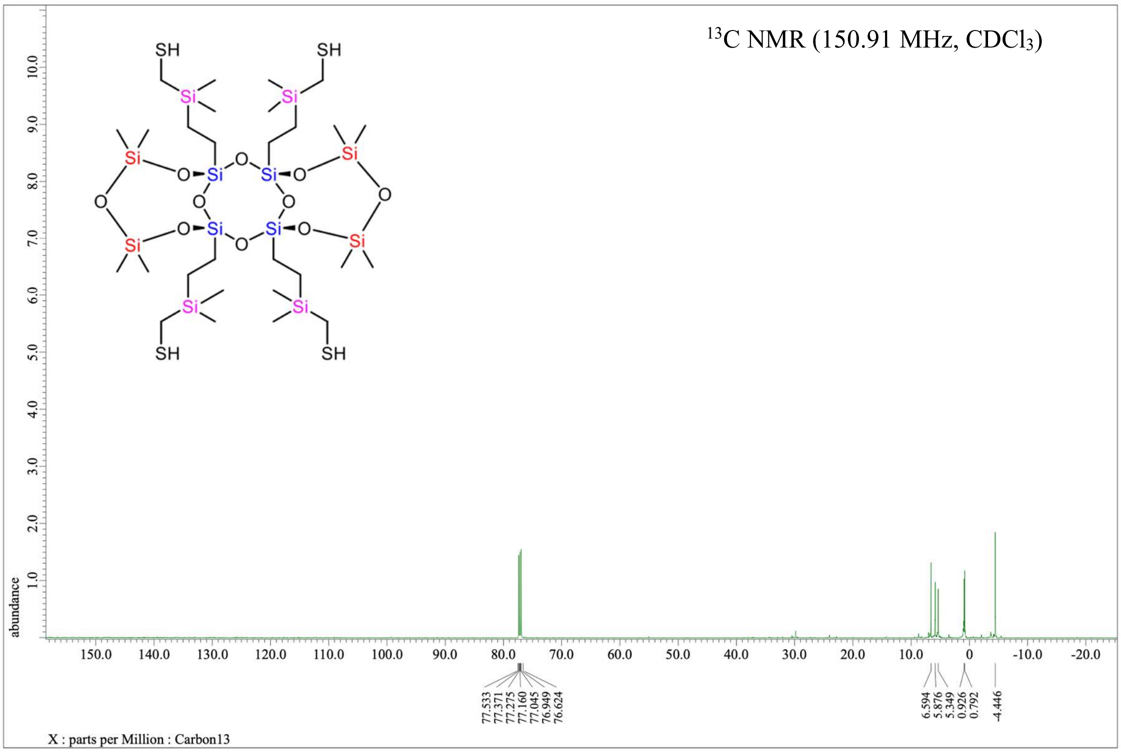

Figure S36: ^{13}C NMR spectrum for compound **9**

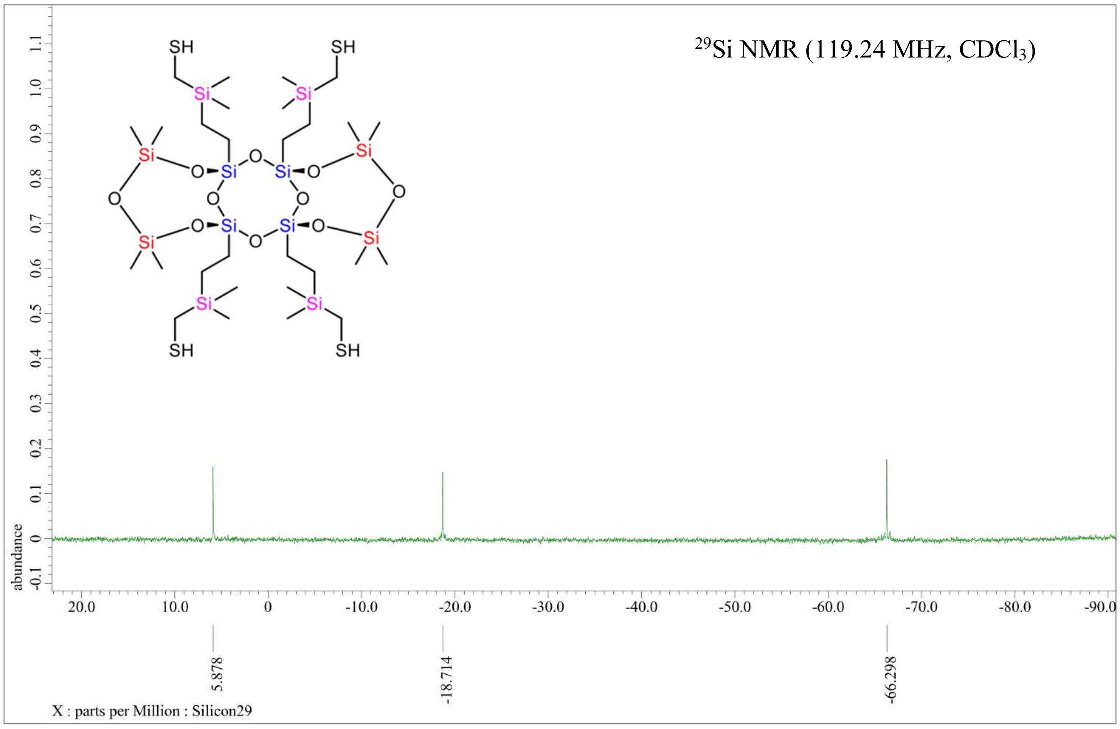

Figure S37: ^{29}Si NMR spectrum for compound **9**

2. MALDI-TOF mass spectra for compounds 2, 4a-4f, 6a, 6b, 8 and 9

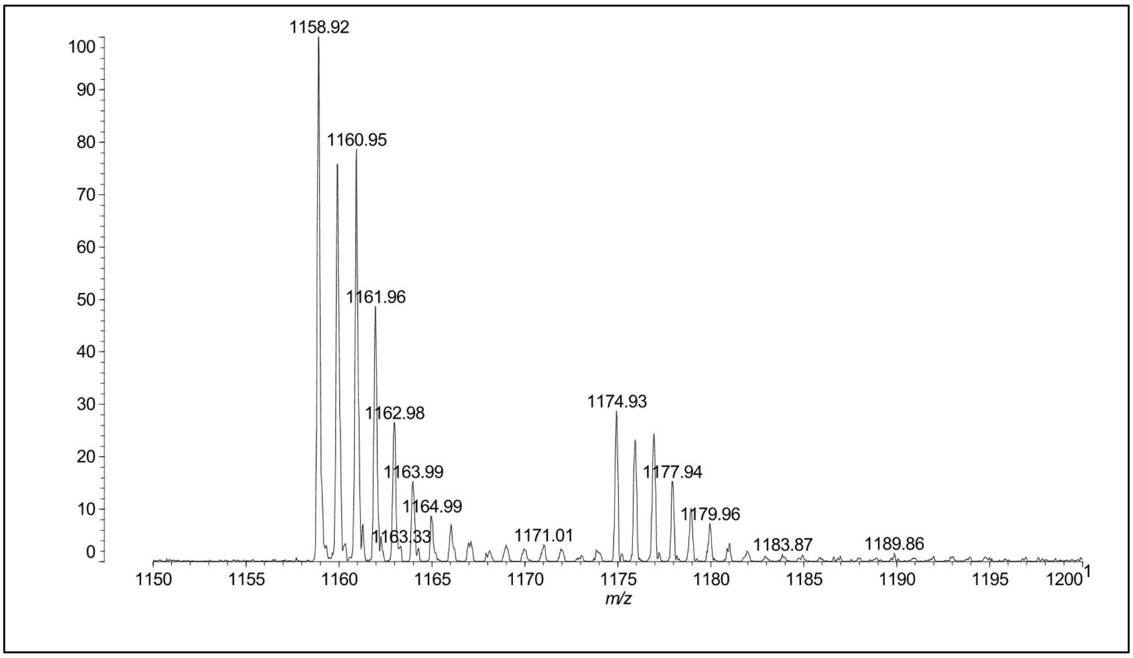

Figure S38: MALDI-TOF analysis for compound **2**

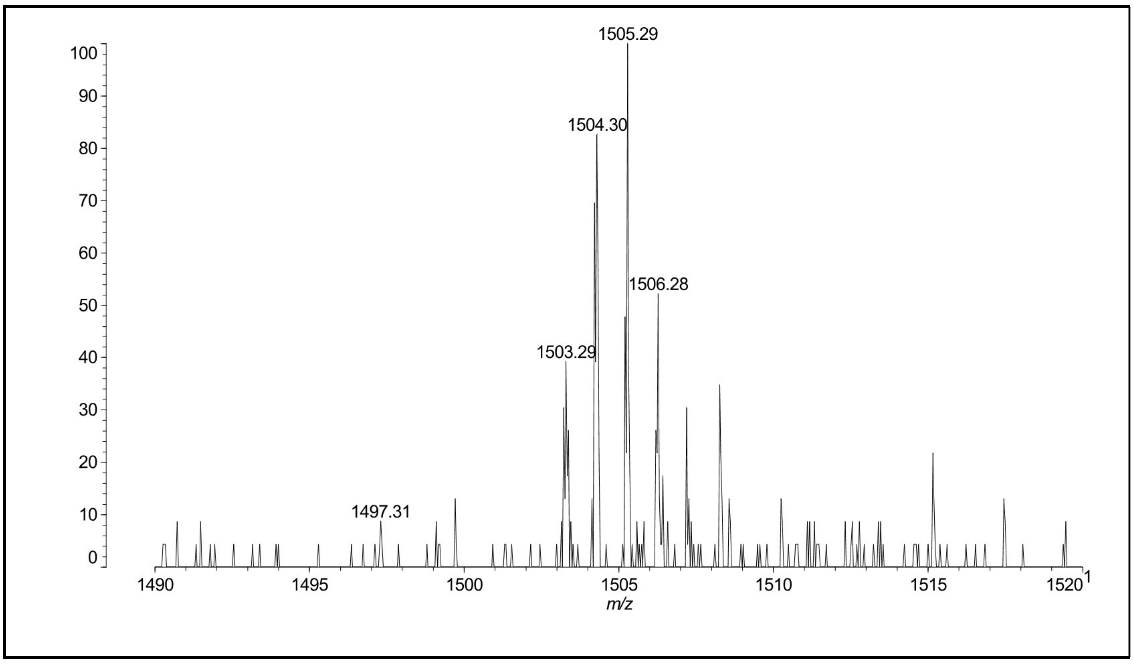

Figure S39: MALDI-TOF analysis for compound **4a**

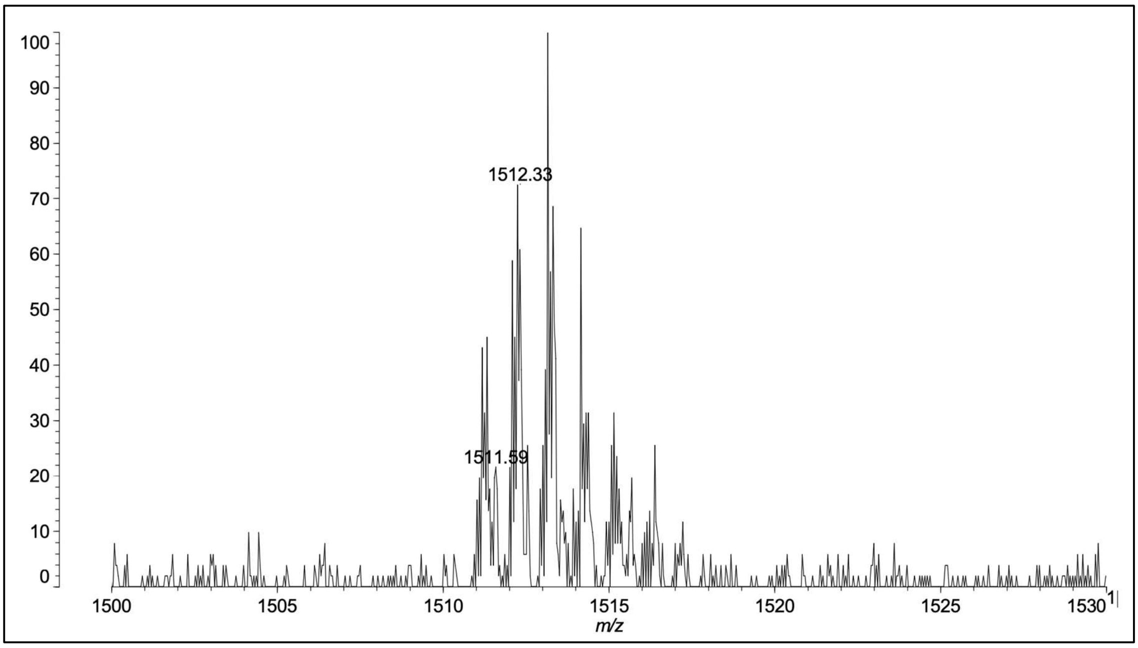

Figure S40: MALDI-TOF analysis for compound **4b**

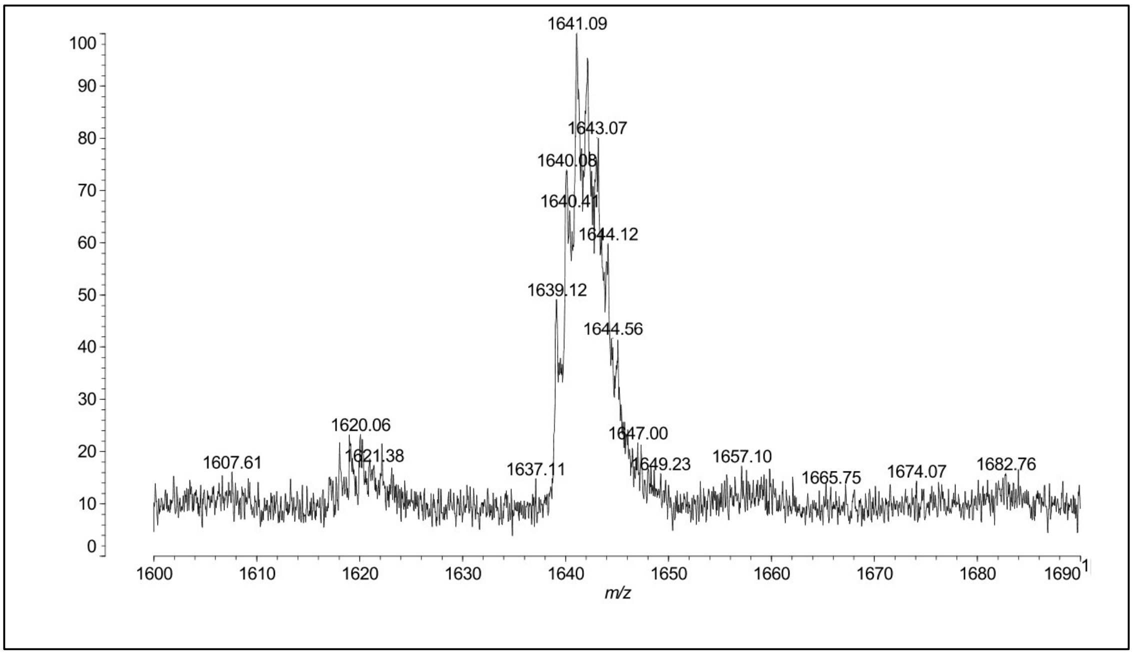

Figure S41: MALDI-TOF analysis for compound **4c**

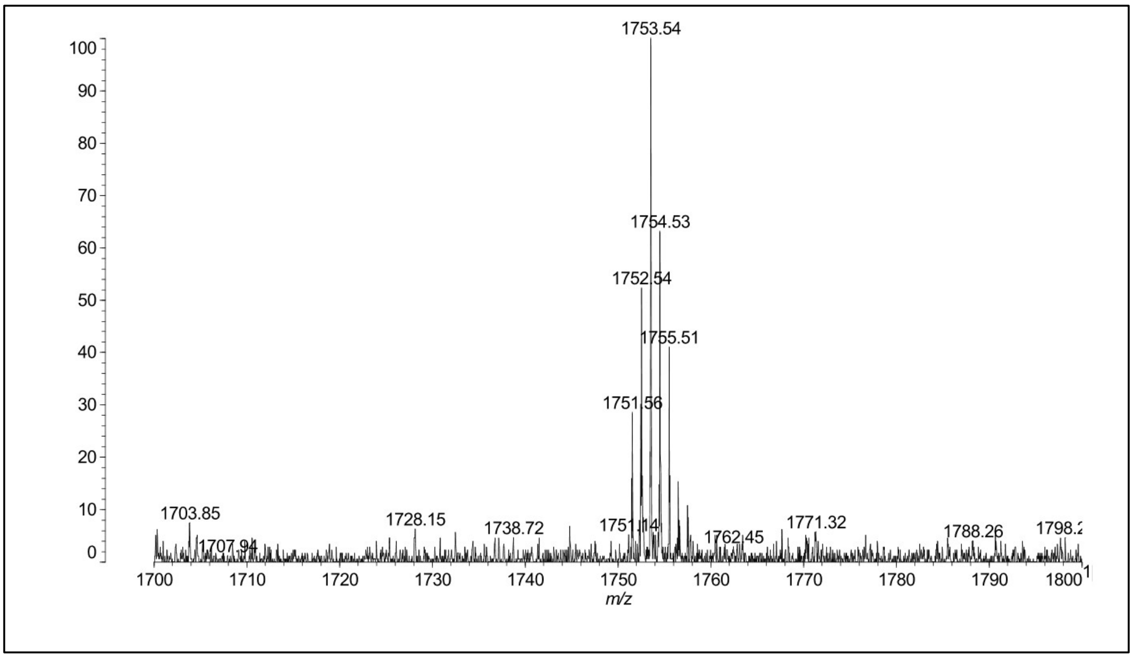

Figure S42: MALDI-TOF analysis for compound **4d**

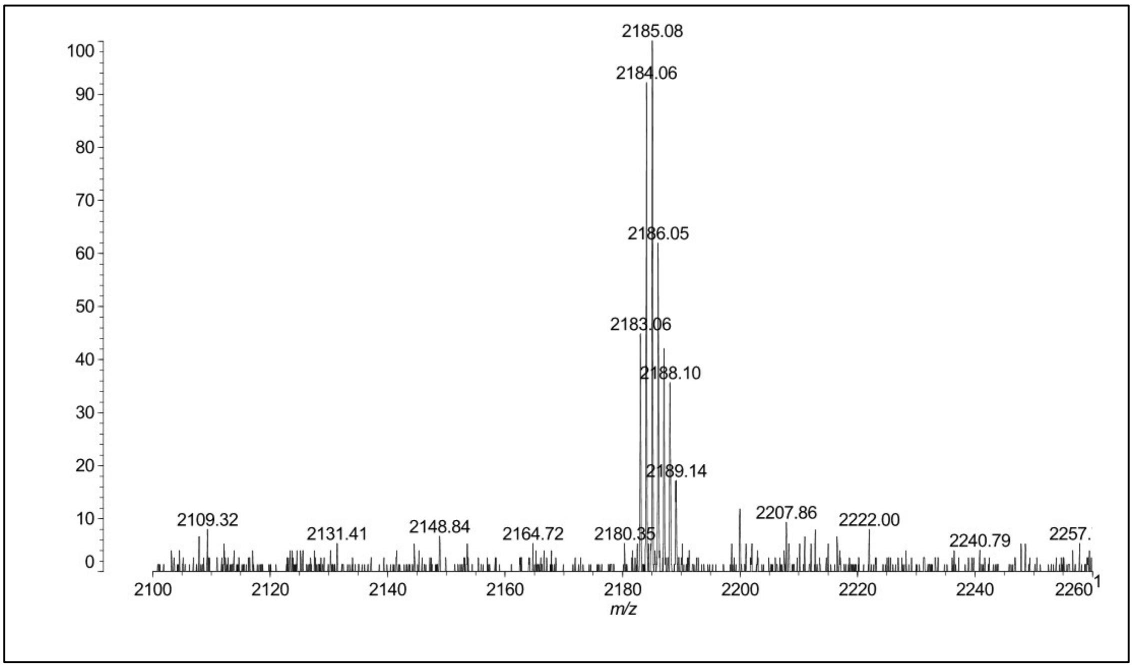

Figure S43: MALDI-TOF analysis for compound **4e**

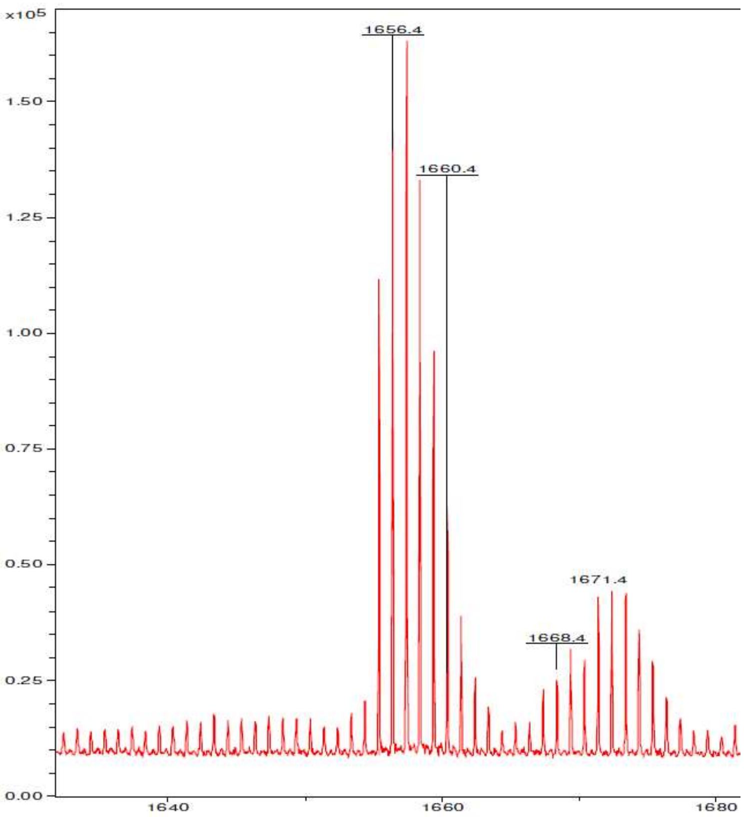

Figure S44: MALDI-TOF spectrum for compound **4f**

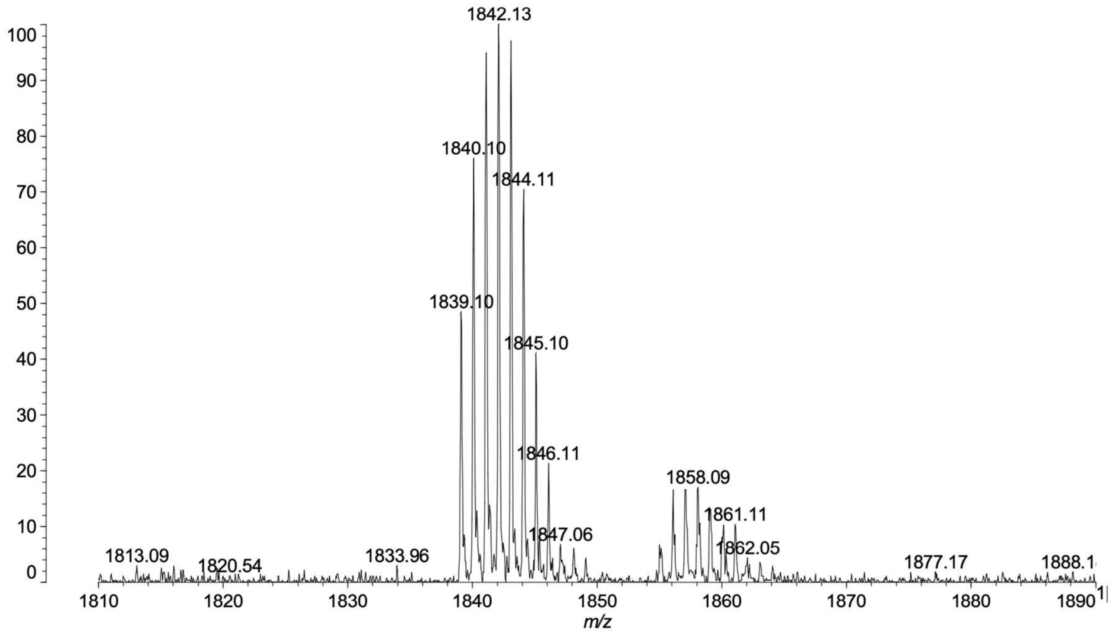

Figure S45: MALDI-TOF analysis for compound **6a**

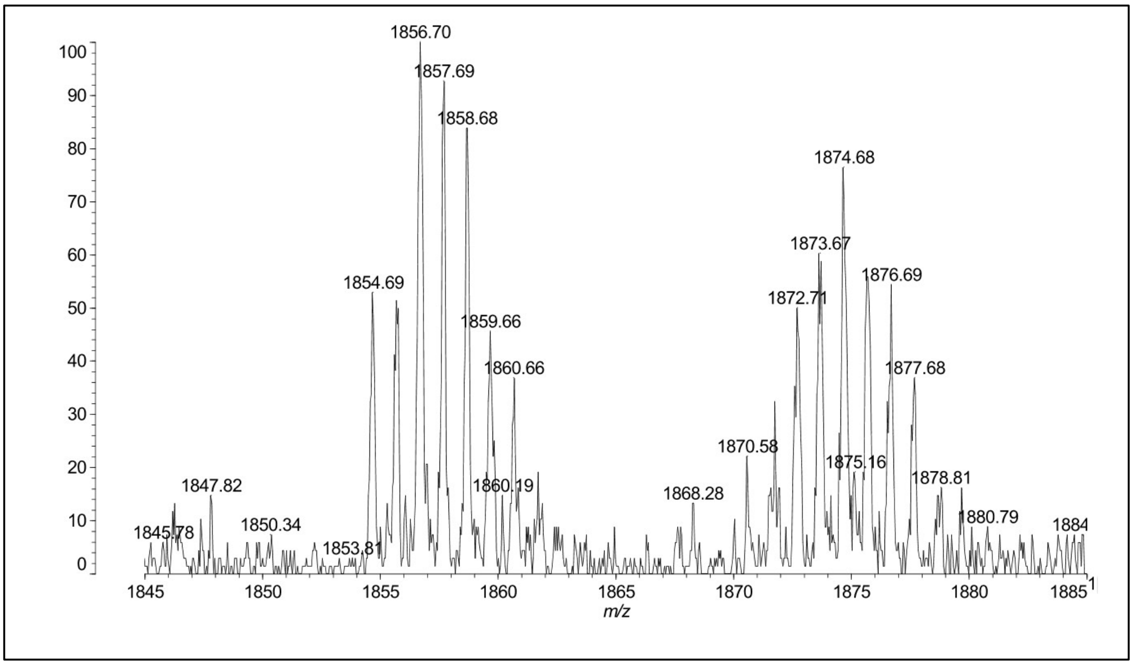

Figure S46: MALDI-TOF analysis for compound **6b**

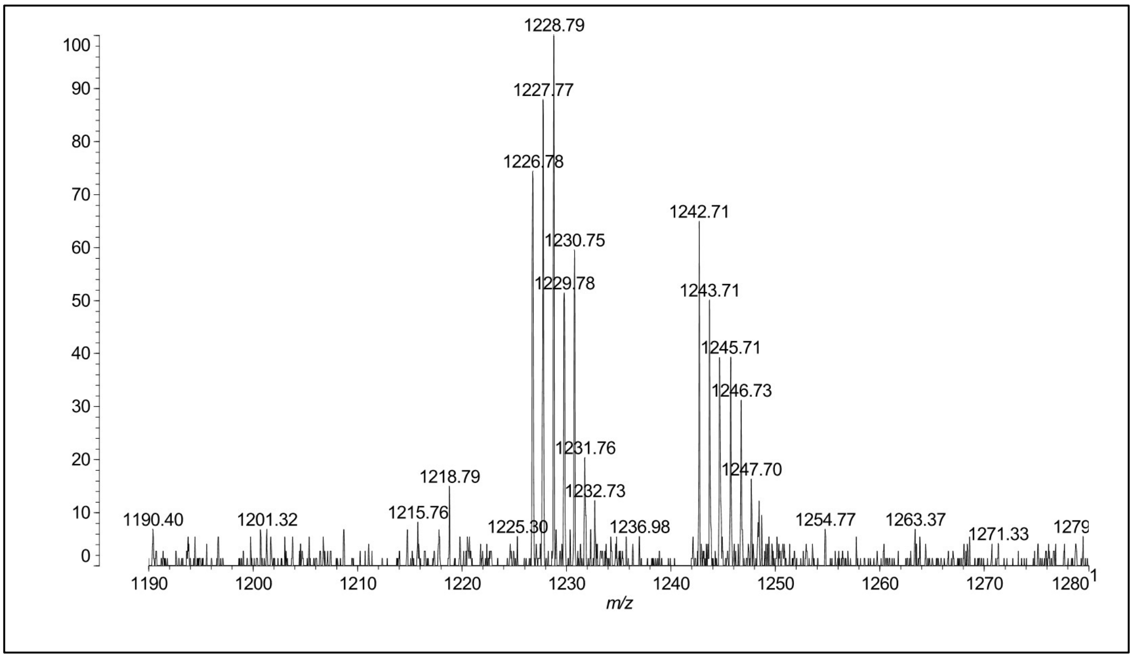

Figure S47: MALDI-TOF analysis for compound **8**

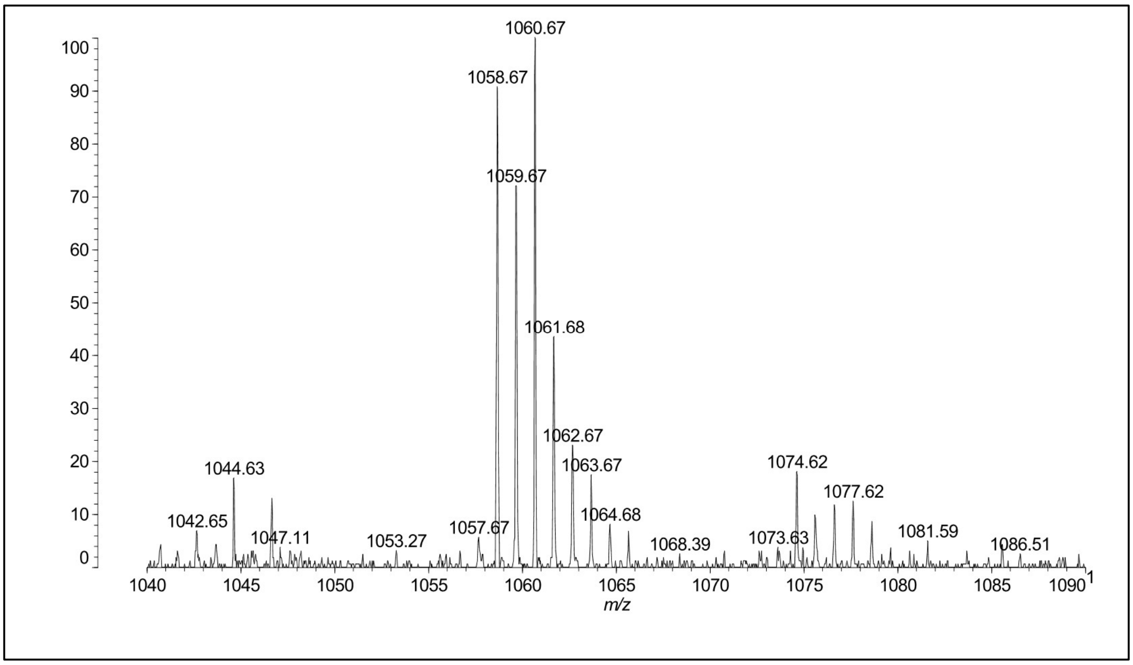

Figure S48: MALDI-TOF analysis for compound **9**

3. Thermogravimetric graphs for compounds 2, 4a-4f, 6a, 6b, 6f, 8 and 9

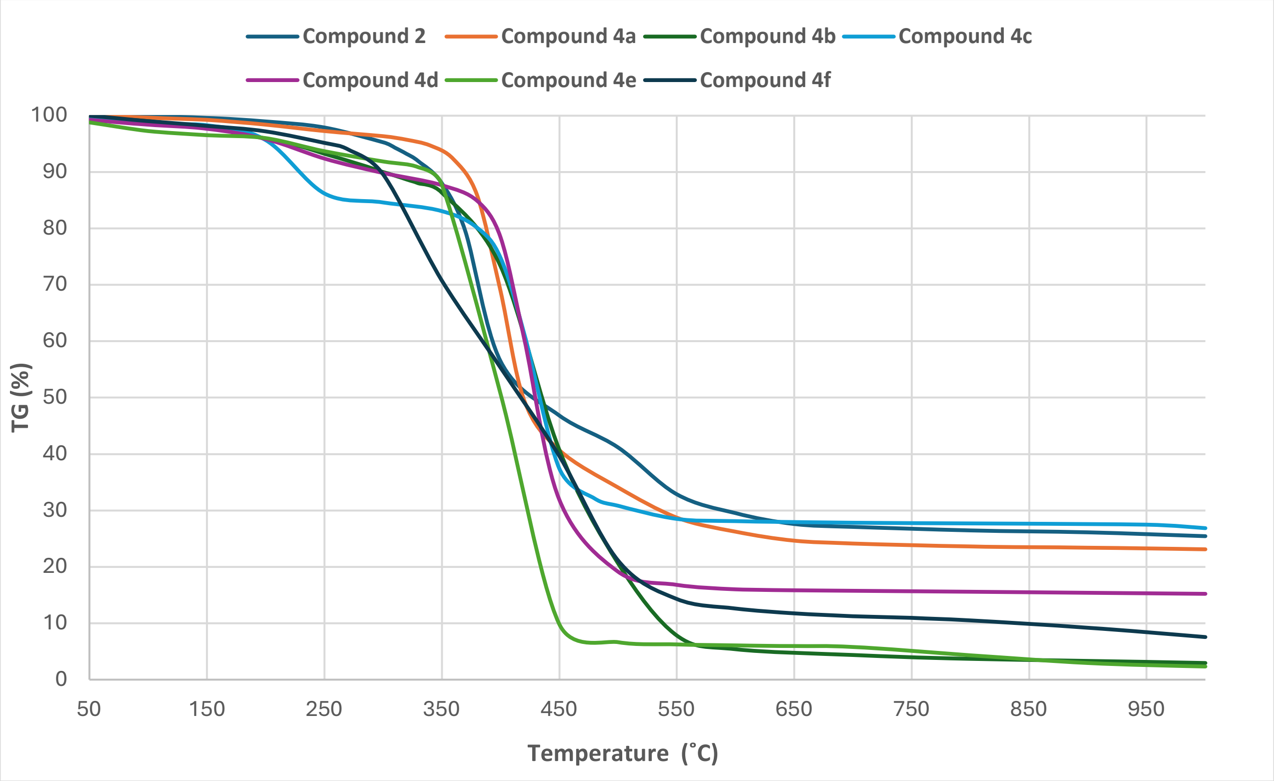

Figure S49: Thermogravimetric graphs for all-cis-T_4 compounds

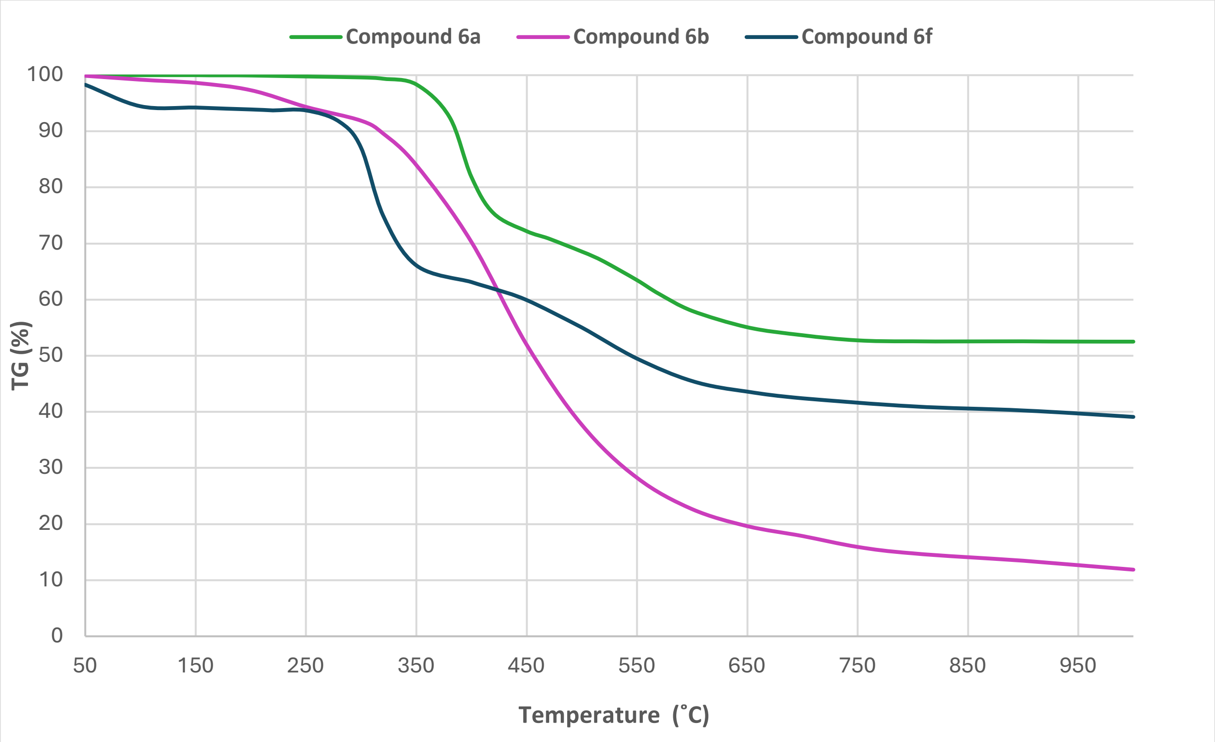

Figure S50: Thermogravimetric graphs for T_8 compounds

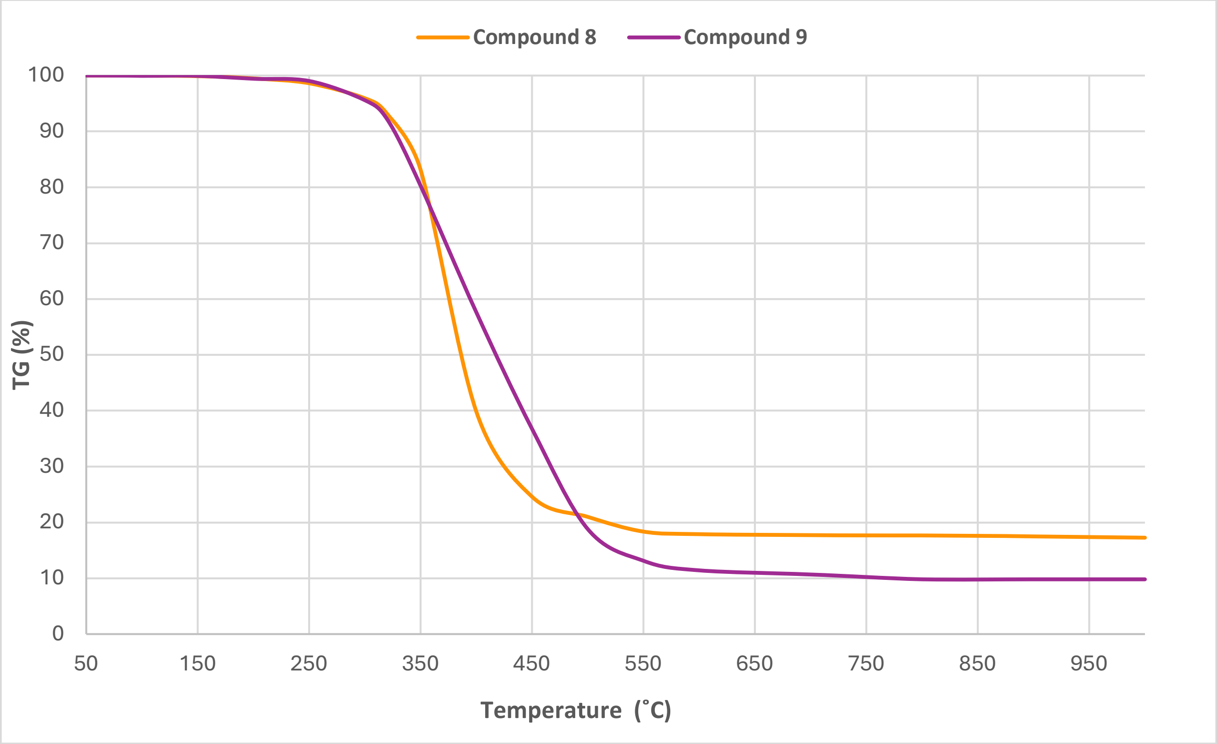

Figure S51: Thermogravimetric graphs for laddersiloxanes
